# My Twenty Years in Microwave Chemistry: From Kitchen Ovens to Microwaves that aren't Microwaves

**DOI:** 10.1002/tcr.201800045

**Published:** 2018-06-15

**Authors:** C. Oliver Kappe

**Affiliations:** ^1^ Institute of Chemistry University of Graz Heinrichstrasse 28, A- 8010 Graz Austria

**Keywords:** microwave chemistry, microwave effects, multimode cavity, single-mode cavity, continuous flow chemistry

## Abstract

This Personal Account describes the author's involvement in the field of microwave‐assisted organic synthesis (MAOS) from the late 1990’s starting out with kitchen microwave ovens right through to the development of a reactor in 2016 that – although not using microwave technology – in many ways mimics the performance of a modern laboratory microwave. The reader is taken along a journey that has spanned two decades of intense research on various aspects of microwave chemistry, and, at the same time, was intimately linked to key innovations regarding equipment design and development. A “behind the scenes” approach is taken in this article to share – from a very personal point of view – how specific projects and research ideas were conceived and developed in my research group, and how in general the field of microwave chemistry has progressed in the last two decades.

## 1 Introduction

When I received the invitation from the Editors of *The Chemical Record* to contribute a Personal Account to an upcoming special issue on microwave chemistry I was initially uncertain how to react. Since my research activities have shifted away from microwave chemistry some time ago, my first instinct was to decline. On second thoughts I found the opportunity to reflect on “my twenty years” in the field of microwave chemistry quite appealing and I ultimately accepted the invitation. This was perhaps motivated, at least in part, by the fact that one of my postdoc advisors, Albert Padwa, had written an inspirational Perspectives Article for *The Journal of Organic Chemistry* in 2009, where he most elegantly reflected on how and why his own research interests had progressed and changed quite significantly during the span of close to 50 years of research in organic synthesis.^1^

The present article is therefore not a standard scientific review but rather a very Personal Account that describes how my research group got involved in specific research topics within the general area of microwave chemistry over a time period of roughly two decades. This somewhat nostalgic journey puts a strong emphasis on *why* things happened the way they did, focusing more on a political “behind the scenes” perspective, and hopefully will be of interest and an entertaining read for the many “microwave chemists” that were active during that time period. As with most Personal Accounts of this nature, the contributions of other scientists in the field will only be mentioned where this is needed to understand the context of our own work. In order not to disrupt the flow of the manuscript too much many of my personal comments have been placed in the reference section and technical jargon is kept to a minimum so the manuscript can be easily followed by the non‐expert reader.

Upon reflecting on the many different turns our research in microwave chemistry has taken over the years I thought it would be most appropriate to divide this article into different sections based on the type of reactor technology that was used at the time. This also allows a more or less chronological and logical discussion of our research.

## 2 The Early Years: From Kitchen Microwaves to Multimode Reactors

### 2.1 Hungary, Rajender S. Varma, and a Beaker Filled with Alumina

My involvement in the field of microwave chemistry started rather abruptly in the summer of 1998 and it was not a planned affair at all. At that time I had just completed the requirements for obtaining my “Habilitation”^2^ at the University of Graz and was looking out for potential new research projects and ideas. My first independent research theme after returning from my postdoctoral stay at Emory University (Atlanta) with Albert Padwa to Graz in May of 1996 was linked to multicomponent reactions, in particular to the three‐component Biginelli dihydropyrimidine synthesis.^3^

As my research involved heterocycles I decided to attend and to present a lecture on my results at a regional conference on heterocyclic chemistry in nearby Hungary.^4^ Opening plenary lecturer at this meeting was Rajender S. Varma, at that time active at Sam Houston State University in Huntsville, Texas, and one of the pioneers in the field of microwave chemistry. Raj Varma presented an inspiring lecture on the use of microwave heating for running synthetic organic chemistry within seconds or minutes instead of hours in the conventional way, most of the times not using any solvent.^5^ At that time, for all practical purposes, the only equipment available to synthetic chemists to run reactions under microwave conditions was a kitchen (domestic) microwave oven.^6^ In the 1990’s, microwave chemistry was still mainly considered a laboratory curiosity and a type of “black box” method, with several theories floating around to rationalize the observed phenomena, which in most cases involved dramatic rate accelerations and sometimes changes in selectivity.^7^ As far as synthetic organic chemistry is concerned, the field got off the ground in 1986 when the first two reports on carrying out organic synthesis in kitchen microwaves were published almost simultaneously by researchers in Canada and the US.^8^ In the late 1990’s, the main players in microwave‐assisted organic synthesis (MAOS), apart from Rajender S. Varma,^5^ were Andre Loupy (France),^6^,^7^ Christopher R. Strauss (Australia),^9^ Antonio de la Hoz, (Spain)^10^ and Ajay K. Bose (USA),^11^ to name only a few.

The lecture by Raj Varma in Hungary was an eye opener for me and I was intrigued by what appeared to be the quite significant potential of this new and enabling technology. I was keen to enter this field as soon as possible and to investigate, as a start, the Biginelli reaction under microwave conditions. I was therefore very pleased that Raj Varma agreed to collaborate with me, so soon after I came back from Hungary I enthusiastically started the first experiments at the University of Graz.^12^ One consequence of using a standard kitchen microwave oven for synthetic chemistry was that one had to run the reactions solvent‐free. The use of solvents, in particular flammable organic solvents, under open vessel microwave conditions (see below) carried the risk of fire, or, if sealed vessels with no control over temperature and pressure were to be used, of explosions.

**Figure 1:**
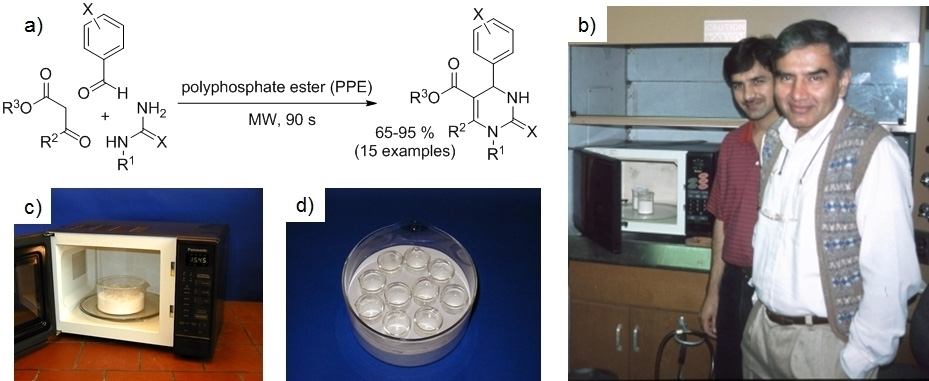
a) Biginelli three‐component reaction under solvent‐free microwave conditions. b) Rajender S. Varma (on the right) and Dalip Kumar in front of their kitchen microwave in Huntsville, Texas on December 8, 1998. c) Panasonic kitchen microwave oven with turntable containing a crystallization dish filled with alumina. d) Detailed view showing 10 beakers (20 mL) inside the alumina bath for performing parallel microwave chemistry.

After considering our options, we decided that the most straightforward approach would be to adapt a protocol for the Biginelli reaction using polyphosphate ester (PPE) as reaction mediator and dehydrating agent that we had published earlier that year using THF as solvent under reflux conditions.^13^ PPE is a non‐volatile substance and therefore appeared perfectly suitable for this purpose. Simply mixing the three components in the Biginelli protocol with an excess of PPE and irradiating them neat under microwave conditions for 90 s provided the desired dihydropyrimidine products (Figure 1a). Unfortunately, things were not as simple as they originally appeared. Despite numerous attempts, I was initially not able to reproduce what Raj Varma and his associates were achieving in their microwave device in Texas. In my case the reaction mixture simply got too hot and violently “moved” out of the reaction vessel. After a frustrating couple of weeks in the lab, I ultimately decided in despair that I needed to pay a visit to Texas to see firsthand how these experiments were done (Figure 1b). After learning that the reaction vessels had to be put in a bath of alumina (acting as a heat sink, Figure 1c and d), I finally was able to obtain good results upon my return and then spent most of the 1998 Christmas holidays in the lab finishing the experimental part of the planned publication.^14^ After getting rebuffed from two organic chemistry journals, my first ever microwave paper ultimately appeared in *Synthesis* in October of 1999.^15^ The most important conclusion for me from this experience was that I would never run another reaction in a kitchen microwave, and I was therefore eagerly searching for alternatives.

### 2.2 The First Laboratory Microwave: The MLS ETHOS System

**Figure 2:**
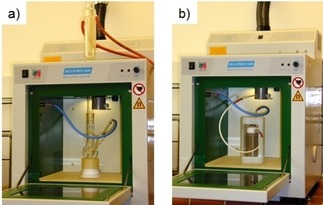
MLS ETHOS 1600 multimode microwave reactor (1999). a) Set‐up for open vessel chemistry under reflux conditions. Note the internal temperature sensor (shielded thermocouple). b) Set‐up for sealed vessel operation using a 100 mL PFA (perfluoroalkoxy polymer) reaction vessel contained in a single high‐pressure PEEK (polyether ether ketone polymer) rotor block segment (including an online pressure sensor).

In mid‐1999, I obtained a grant from an Austrian funding institution to purchase a dedicated microwave reactor with proper temperature and microwave power control, also featuring magnetic stirring.^16^ As the actual grant was reduced by almost 90 % compared to what I had applied for, the best deal I could make at the time was to obtain a demo unit (with limited warranty) of a newly developed multimode laboratory microwave reactor from MLS GmbH in Germany (MLS ETHOS 1600, Figure 2).^17^ At about the same time my first PhD student, Alexander Stadler, joined the group (October 1999), funded by my still active grant on dihydropyrimidine chemistry from my Habilitation years.^18^ We immediately set‐out to evaluate how the Biginelli reaction (*cf*. Figure 1a) would run in a solvent such as ethanol containing catalytic amounts of HCl under controlled microwave conditions, i. e., applying the internal temperature measurement system (Figure 2a). Much to our surprise (at that time), the results obtained by heating the reaction mixture at reflux to 80 °C in an oil bath for 3 h were virtually identical to what could be achieved under reflux microwave conditions at the same temperature.^19^ Evidently, exposing the reaction mixture to 2.45 GHz microwave irradiation under reflux conditions had no direct effect on the chemistry, apart from heating the reaction mixture. Since our originally purchased multimode unit did not offer the capabilities to run experiments at a higher reaction temperature in a sealed vessel with online pressure control, we had to borrow the appropriate 100 mL reactor and a pressure sensor (Figure 2b) from our colleagues in analytical chemistry, who routinely used sealed vessel microwave technology and this instrument configuration for sample preparation purposes (a very well‐known technique called microwave digestion that was already state‐of‐the art at the time in the analytical chemistry community).^20^ Raising the temperature of the reaction mixture to 180 °C (20 bar pressure) unfortunately provided considerably reduced product yields and a number of unwanted side products.^19^ The previously published dramatic rate enhancements in the Biginelli reaction using the ethanol/HCl system (3–6 min reaction time)^21^ could only be reproduced, ironically, when a kitchen microwave was used – as described in the original reference^21^ – with the reaction mixture being irradiated in a standard beaker. Under these – quite hazardous – processing conditions the solvent rapidly evaporates and a therefore much more concentrated reaction mixture is ultimately processed. This leads to more rapid conversions and higher yields – an effect that we could duplicate by conventional heating and was thus not directly related to the use of microwave irradiation.^19^

While I perhaps had expected a somewhat more “exciting” outcome from our first study using a dedicated microwave reactor, the MLS multimode instrument has subsequently been utilized in our group for many years, mainly in those instances where other microwave instruments that we acquired (see below) could not be used, i. e., for open vessel processing (involving solid‐phase organic synthesis in single and multiple vessels),^22^,^23^ microwave‐assisted fractional distillations,^24^ and in cases where the heating performance of various objects placed inside a microwave cavity needed to be evaluated.^25^

The main limitation of the MLS ETHOS system was, however, that it was not designed for small scale (<5 mL) organic synthesis, but had been adapted from a microwave digestion unit. In microwave digestion the reaction mixture, typically a Brønsted acid, is almost always strongly microwave absorbing.^20^ In organic synthesis it is not uncommon that the reaction medium is of a nonpolar nature and will therefore not absorb microwave energy very well. In such instances, other materials present in the microwave cavity, i. e., a metal thermocouple, or even certain polymeric materials can interact with the microwave field.

## 3 The Single Mode Revolution

### 3.1 The Smith Synthesizer, Coherent Synthesis and Personal Chemistry

Early in the year 2000, I heard rumors that a company from Sweden would launch a new revolutionary microwave synthesizer. I knew that a group at Uppsala University (Anders Hallberg and Mats Larhed) had published a series of articles since 1996 on high‐speed microwave chemistry involving transition‐metal catalyzed transformations.^26^ The instrument they used was a so‐called single‐mode reactor design^27^,^28^ called MicroWell 10 from Labwell AB in Uppsala.^29^ Experiments were performed on relatively small scale (≤5 mL) in sealed glass vessels and – although the instrument did not have an algorithm to control the reaction temperature by modulating microwave power output like the MLS system we used – impressive results were obtained, reducing reaction times for many different transformations from hours to minutes, often improving yields at the same time.^26^

**Figure 3:**
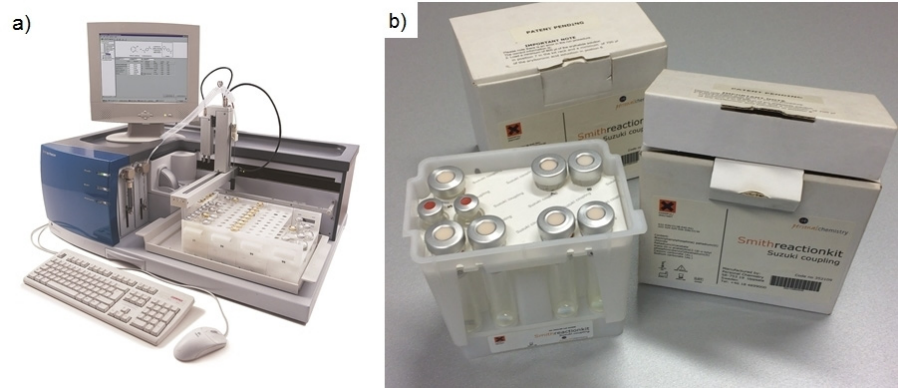
a) Personal Chemistry Smith Synthesizer (2000). b) Smith Reaction Kit (the Suzuki coupling kit is shown) containing pre‐dispensed catalysts, reagents and additives. Reproduced with permission from ref. 28b.

**Figure 4:**
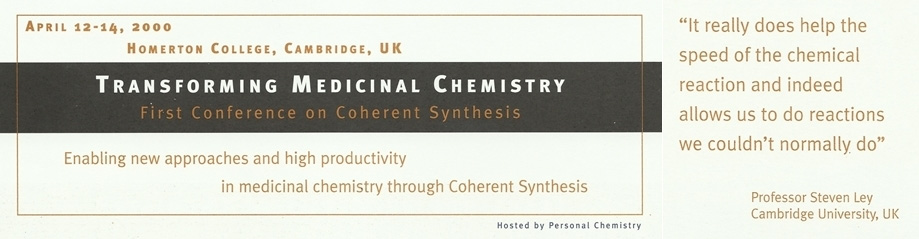
Conference Brochure from the Coherent Synthesis Conference in April of 2000 in Cambridge. The keynote lecture was given by Steven V. Ley.

The mysterious new system, initially called “Smith Synthesizer” (Figure 3a), was ultimately launched by Personal Chemistry AB (formally Labwell AB) in April of 2000 at a special event in Cambridge that I happened to attended (Figure 4).^30^ The instrument used 10 mL borosilicate glass vessels that could be sealed within seconds by a PTFE septum and an aluminum crimp top (much like an HPLC vial) allowing operation up to 20 bar pressure at a maximum temperature of 250 °C. Vials were moved in and out of the microwave cavity in an automated fashion by a gripper with reagents either being filled manually into the vials before capping, or dispensed through the PTFE septum via an incorporated liquid handler. Using the liquid handler option, a sequence of reagent additions from, e. g., different stock solutions into different process vials could be programmed and executed in an automated and completely unattended fashion. Importantly, compared to the MicroWell 10, the Smith Synthesizer had an external infrared temperature sensor measuring temperature on the outer surface of the process vial. An algorithm would regulate the microwave output power in such a way that the preselected maximum temperature would be maintained for the desired reaction/irradiation time. Compressed air was used to quickly cool down the samples after the heating cycle was completed, a design feature incorporated by all other single‐mode microwave devices that were subsequently developed (see below).

The vision of Personal Chemistry however did go far beyond microwave chemistry itself. The philosophy under which the system was marketed was called Coherent Synthesis and incorporated reaction planning systems, automation, distributed system access, searchable databases and reaction optimization kits.^31^ So‐called Smith Reaction Kits (Figure 3b) contained pre‐dispensed reagents, catalysts, etc. for the most common transformations in organic synthesis (e. g., Suzuki cross‐couplings, Heck reactions, various protection and deprotection protocols). The design and development of such a kit involved the running of hundreds of reactions using chemometric experimental design and optimization routines to result in a small set of standardized reaction conditions that suit most substrates. A database that was implemented at the same time provided access to thousands of microwave reactions, including details of procedures, analytical results, etc.^31^

Clearly, the microwave system and overall concept was designed having the pharmaceutical industry and drug discovery in mind. Nevertheless, the Smith Synthesizer appeared extremely attractive to me as it would allow us to run synthetic transformations on small scale (up to 5 mL reaction volume) under controlled temperature conditions and using some degree of automation. The hefty price tag made it obvious that no academic group could ever afford such an instrument.^31^

In September of 2000, along with my PhD student Alexander Stadler, I attended the 2^nd^ International Conference on Microwave Chemistry, in Antibes/Juan‐les‐Pins on the French Riviera and we both presented the results from our recently published work.^19^,^22^ During the conference I learned more details about Personal Chemistry and the Coherent Synthesis concept from Åke Pino Pilotti, Professor at Stockholm University, but also the coordinator of Personal Chemistry's Scientific Partnership Program. After some discussions in the weeks and months that followed our group in Graz was officially made part of Personal Chemistry's Scientific Partnership Program in January of 2001, which not only involved getting a Smith Synthesizer into my lab (ultimately installed in March of 2001), but also a supply of consumables and funding for a PhD student. In return, the ensuing results from our microwave experiments conducted on the system were entered into a reaction database to allow the Coherent Synthesis concept to work and thus enhancing the growth of database content.

In order to evaluate the automation and robotic features of the Smith Synthesizer to their full potential we first set out to synthesize a library of dihydropyrimidines using – again – the venerable Biginelli reaction as model transformation utilizing ethanol as a solvent (*cf*. Figure 1a). In contrast to the open vessel experiments at 80 °C described in Section 2.2. careful optimization under sealed vessel conditions now revealed that at 120 °C (∼5 bar) and in the presence of a Lewis acid catalyst most transformations could be completed within 10 min providing high product yields. In contrast to a traditional parallel library approach each of the library compounds could now be processed individually (applying varying processing conditions and reagent/catalyst concentrations if necessary). Using a diverse set of CH‐acidic carbonyl compounds, aldehydes, and urea/thiourea derivatives a representative sub‐set of 48 dihydropyrimidine analogs was prepared using automated addition of building blocks and subsequent sequential microwave processing of each process vial. The results of these studies were published in the November/December 2001 issue of the *Journal of Combinatorial Chemistry*.^32^

This was the first in a series of many publications from my laboratory using the Smith Synthesizer and the microwave instruments from Personal Chemistry/Biotage that were developed in subsequent years.^33^ Being an academic lab, we did not use the liquid dispensing mode too many times but the gripper moving vessels in an out of the cavity was a useful feature allowing unattended processing of many samples in a row.

### 3.2 High Speed Organic Synthesis and Combichem Applications

**Scheme 1:**
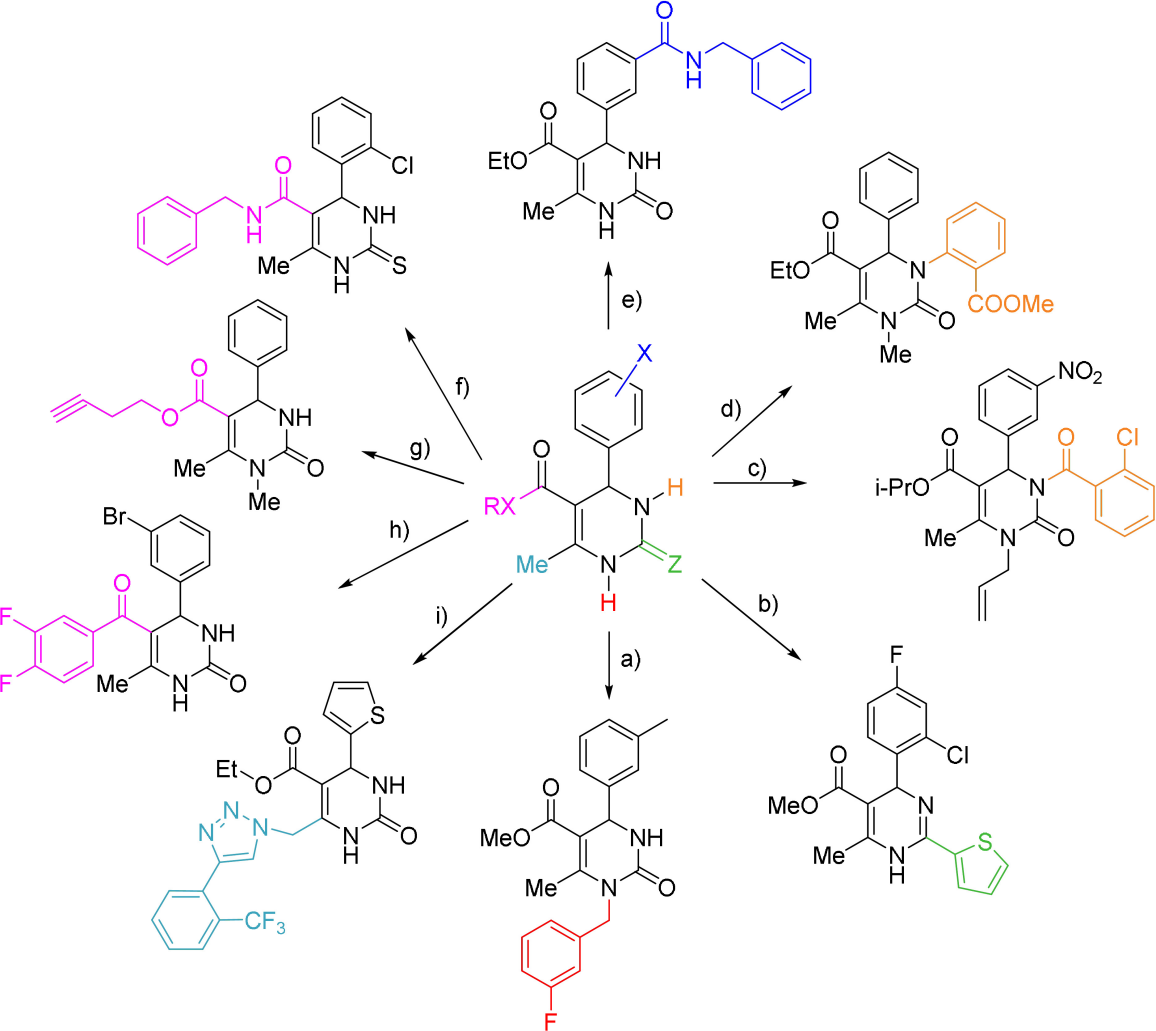
Microwave‐assisted scaffold decoration on dihydropyrimidines employing controlled microwave heating in dedicated single‐mode reactors. One selected example from each of the 9 sublibraries synthesized is shown. a) Mitsunobu N1 alkylation, b) Pd‐catalyzed Liebeskind‐Srogl type reaction (Z<C=>S), c) N3 acylation with acid chlorides or anhydrides, d) Cu‐catalyzed Goldberg N3 arylation, e) Pd‐catalyzed aminocarbonylation (X=Br), f) amide bond formation (RX=OH), g) Mitsunobu alkylation (RX=OH), h) Pd‐catalyzed Liebeskind‐Srogl reaction (RX=EtS), i) Cu‐catalyzed azide‐alkyne cycloaddition (following C6 bromination and displacement with azide).

All through my early career and particular during my Habilitation years, I maintained an interest in combinatorial chemistry applications, including solid‐phase synthesis, the use of polymer‐supported reagents, parallel synthesis, multicomponent reactions, etc.^34^ In the early 2000’s combinatorial chemistry was still a hot topic and the first papers started to appear that involved both microwave technology and combinatorial chemistry principles.^35^ After being appointed Associate Professor at the University of Graz in March of 2000^2^ and getting to the tail end of my grant on dihydropyrimidine chemistry,^18^ I was eager to obtain some funding for our microwave chemistry program at the University of Graz. Unfortunately, it took several attempts and two and a half years to finally receive public funding for our planned projects linking microwave technology with combinatorial chemistry principles.^36^ A new graduate student, Doris Dallinger, started her PhD in late 2001 and subsequently developed numerous high‐speed microwave protocols related to the scaffold decoration of dihydropyrimidines. Scheme 1 highlights some of the microwave‐assisted transformations developed during those years.^37^ With the exception of the Mitsunobu protocols (1a and g), all transformations worked exceedingly well in the elevated temperature regimes prevalent using sealed vessel microwave processing.

During the period 2002 till 2005, we were able to demonstrate and confirm many of the now well‐known advantages of using controlled microwave heating in organic synthesis – such as faster reactions, cleaner processes and sometimes improved product yields, etc. – for a large variety of different chemical processes. These transformations included, in particular, transition‐metal catalyzed cross‐coupling reactions (some examples are shown in Scheme 1), but also involved solid‐phase organic synthesis, the use of polymer‐supported reagents/scavengers and fluorous reagents, more examples of multicomponent reactions, cycloadditions and many other transformations.^38^ Most of the experiments were performed using our (in the meantime two) single‐mode reactors from Personal Chemistry/Biotage, which were the workhorse of the group during that time. Certainly not only in our lab, microwave heating became “first choice and not last resort” for organic synthesis, and the technology was also applied to several multi‐step syntheses, with each of the steps performed using controlled microwave heating.^39^ At that time we had essentially stopped doing comparison experiments between microwave and conventional processing when the focus of the project was to get to the final compound(s) of interest or to develop new synthetic methodology.

In these early years, I was fortunate to have, in addition to my own very dedicated PhD and master students, a group of talented postdocs, exchange students, and visitors in the laboratory that often provided their own funding. This increased our research output significantly.^38^ In addition, successful academic collaborations were started with the groups of Erik Van der Eycken (Leuven), Mats Larhed (Uppsala), and Romano V. A. Orru (Amsterdam), among others.

### 3.3 A Brief Multimode Interlude: Anton Paar and the Synthos 3000

During the year 2002, I was contacted by a local company in Graz called Anton Paar. Although I had heard of the company, I was not aware of the fact that Anton Paar had been active for many years in the microwave digestion field and had developed multimode instruments for that purpose (not unlike MLS/Milestone and several other companies).^20^ After sorting out our contractual obligations with Personal Chemistry, a loan of one of their multimode instruments was arranged, and in our first joint project the scale‐up of single‐mode (5 mL) to multimode (≤500 mL) microwave chemistry was investigated, performing a side‐by‐side comparison between the two instruments. In all of the 6 cases investigated, it was possible to achieve similar isolated product yields on going from small scale to larger scale (using an eight vessel rotor system) without changing the previously optimized reaction conditions.^40^ A particular advantage of this reactor was that reaction vessels could be pre‐pressurized with reactive gases (not possible in the single‐mode reactors from Personal Chemistry/Biotage), a technique that was subsequently explored in more detail for cycloaddition reactions involving ethylene gas at 10 bar pressure.^41^

**Figure 5:**
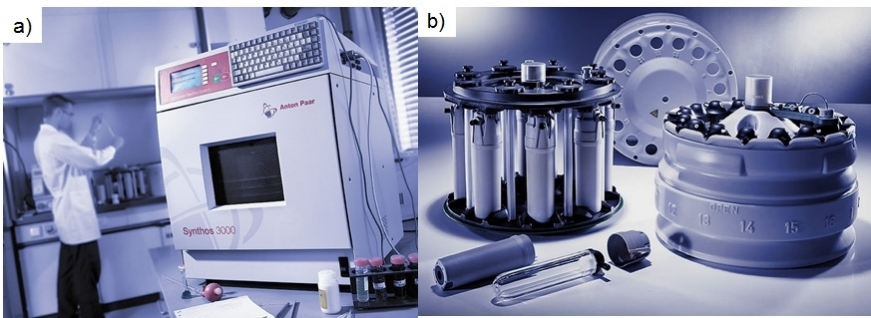
a) Anton Paar Synthos 3000 multimode instrument (2004). b) 8 (left) and 16 (right) vessel rotor systems with a 100 mL quartz vessel pictured in front. Reproduced with permission from ref. 28b.

The success of these early trials ultimately led to the launch of a multimode reactor specifically designed for larger scale organic synthesis named Synthos 3000 by Anton Paar in October of 2004 (Figure 5).^42^ At the same time, a more formal collaboration with Anton Paar was initiated, and a new PhD student, Jennifer M. Kremsner, started work in January of 2004. In one of her first projects, we explored the ability to access water under near‐critical conditions (300 °C, 80 bar) by using specially designed quartz vessels in the Synthos 3000 reactor.^43^ In addition to my passion for microwave chemistry in those days, early initiatives in flow chemistry started in the lab at about the same time.^44^,^45^

## 4 The Heydays of Microwave Chemistry, Gartners Hype Cycle and Frequent Traveling

In my opinion, the “single‐mode revolution” of 2000 has introduced what I call the “golden decade” of microwave‐assisted organic synthesis (2001–2010). The benefits of controlled microwave heating quickly became evident not only to synthetic organic chemists but in particular also to the medicinal chemistry/drug discovery community as outlined in a 2001 review article by Mats Larhed and Anders Hallberg.^46^ A commentary by Paul Edwards from Pfizer published the same year entitled “More microwave reactors required” argued that both industry and academia quickly needed access to more single‐mode microwave reactors in order be able to develop more high‐speed chemistries.^47^

**Figure 6:**
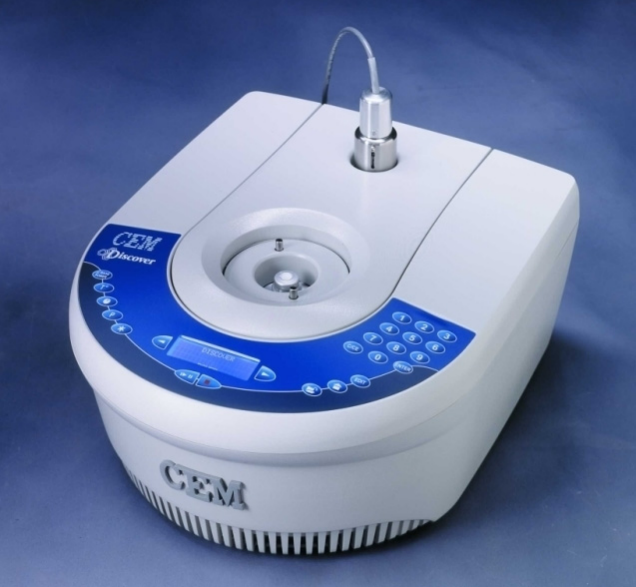
Single‐mode CEM Discover (2001). Reproduced with permission from ref. 28b.

In March of 2001, US‐based CEM Corporation introduced a new type of single‐mode microwave reactor named Discover. Similar to MLS/Milestone and Anton Paar, CEM had been active for many years in microwave digestion and related fields mainly using multimode instruments.^20^ In sharp contrast to Personal Chemistry's philosophy, however, their first instrument was a stand‐alone reactor that could be operated without a PC (Figure 6), but otherwise was quite similar to the Smith Synthesizer with respect to its operating limits, vessel sealing technology, temperature measurement and control algorithms, and post‐reaction cooling by compressed air.^48^ Notably, the CEM Discover allowed also open vessel processing conditions, i. e., performing reactions under reflux. As one could have predicted, the instrument proved very popular in the academic community owing to its significantly lower price tag compared to the Smith Synthesizer from Personal Chemistry.^49^

With the entry of CEM in 2001 and Anton Paar in 2004, there were now four microwave equipment vendors on the market that served the organic synthesis/medicinal chemistry communities, with applications subsequently extending into solid‐phase peptide synthesis, nanoparticle generation, polymer chemistry and many more related scientific disciplines. A market survey released in 2005 predicted a 15–20 % growth rate of the instrument market for synthesis and also forecasted that within 5–6 years the chemical synthesis segment would overtake the analytical segment.^50^ One can imagine the fierce competition that started between these four companies promoting the specific benefits of either their single‐mode or multimode instruments, both in traditional markets such as Europe, North America and Japan, but also on a global scale in emerging markets.^51^

**Figure 7:**
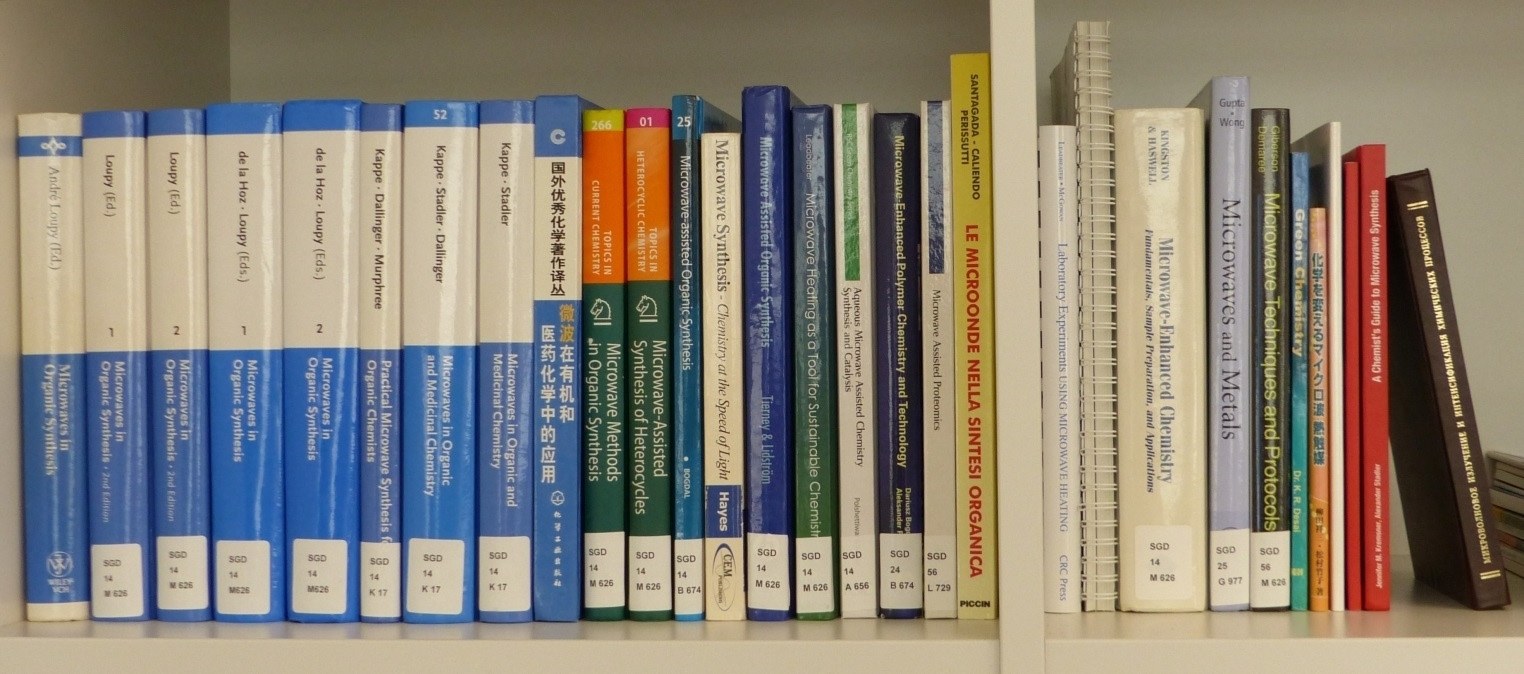
An image of my collection microwave chemistry books.

With more and more academic groups getting involved in microwave chemistry, the number of publications started to skyrocket. Starting in 2005, we introduced a monthly lunchtime journal club where each member of the group – being responsible for one or more publishing houses – presented selected examples of MAOS from the recently published literature. Notably, only those publications where controlled microwave chemistry experiments involving dedicated equipment with online temperature monitoring had been reported were included.^52^ In 2008, the annual number of publications was getting close to 1000 (compared to less than 70 in 2002 and 270 in 2004), and since our routine involved full text searches and was therefore rather time consuming, we were forced to stop at this point.^53^ Journals started to organize special issues on microwave chemistry, in parallel an enormous number of review and feature articles appeared in the literature.^54^ Book publishers were continuously pushing academics for new titles on microwave chemistry (Figure 7).

On the Gartner hype curve, I believe that the “peak of inflated expectations” in microwave chemistry was reached somewhere between 2003 and 2005. *Nature* was publishing a news feature on microwave chemistry in 2003, *Chemical & Engineering News* and *Chemistry World* followed with articles in 2004 and in subsequent years many similar news stories touting the benefits of MAOS appeared in print and online.^55^ At that point, practically any organic transformation and name reaction that tolerated heat had probably been tried in a microwave system by somebody, and, quite remarkably, even some of those transformations that were assumed not to tolerate any heating, like solid‐phase peptide couplings, were successfully executed using microwave conditions.^56^ Under the spirit of “anything goes” prevalent in those days the scientific community was even led to believe, at least for a while, that Suzuki cross‐coupling reactions performed under microwave conditions could proceed without any transition metal.^57^

**Figure 8:**
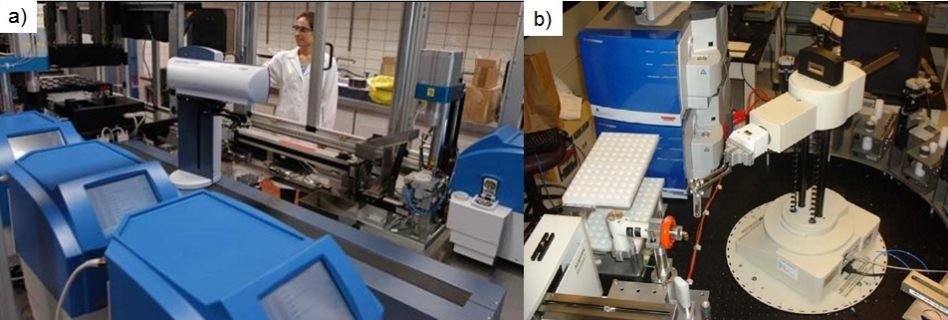
a) Novartis (Basel, Switzerland) high‐throughput microwave synthesis factory. b) Abbott Laboratories (Illinois) robotic microwave facility. Reproduced with permission from ref. 28b.

Parallel to what happened on the academic front, pharma massively invested in the technology as well following to some extent the Coherent Synthesis philosophy. In order to speed up their drug discovery operations facilities containing multiple microwave reactors were installed and coupled with suitable robotics, automation, and, in particular, work‐up and purification strategies on the back‐end (Figure 8). Given the benefits of speed, reproducibility and automation, coupled to the fact that the average size of compound libraries was coming down significantly at that time, the concept of automated sequential library synthesis under controlled microwave conditions (as opposed to the standard parallel synthesis approach) appeared very attractive.^46^,^58^,^59^

**Figure 9:**
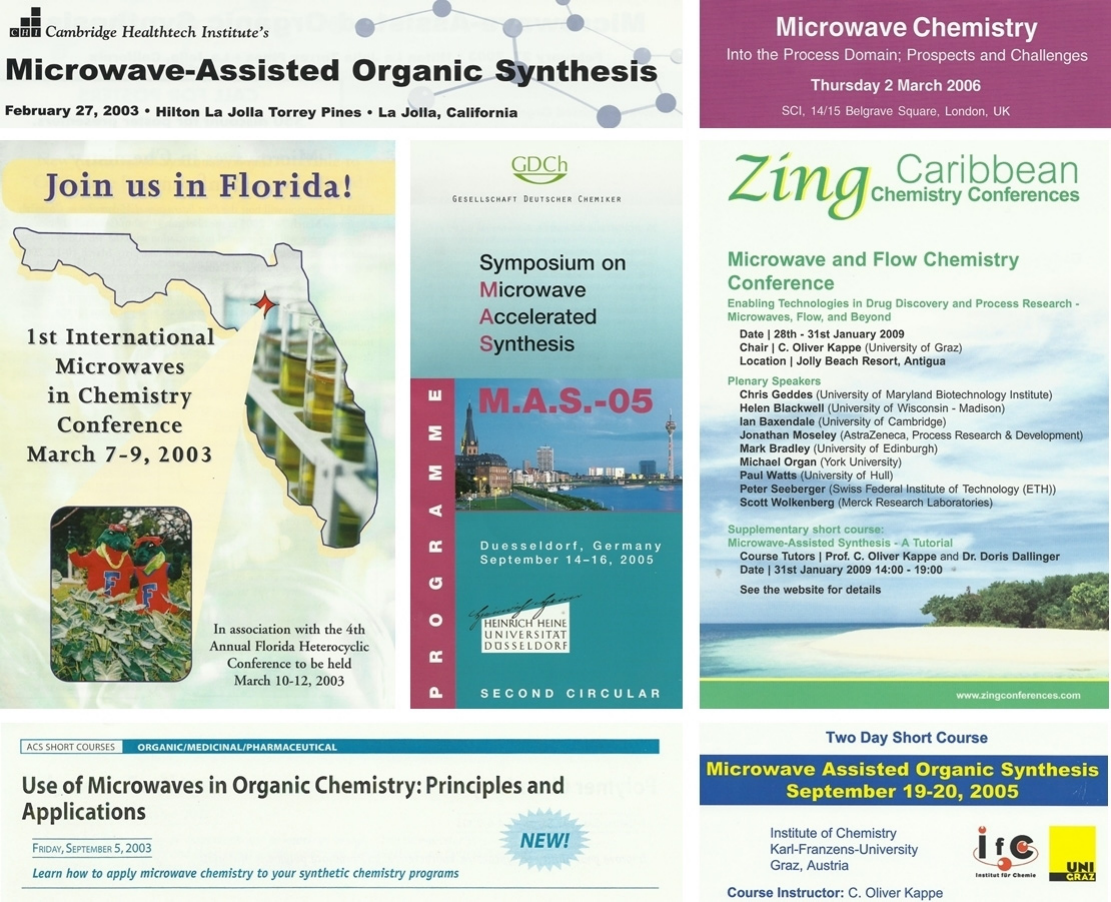
A selection of conference brochures from microwave chemistry conferences and short courses held between 2003 and 2009.

On top of all the scientific and publishing activities, conferences started to emerge that specifically addressed the microwave chemistry community. In the beginning, the microwave theme was often part of sessions on enabling technologies in medicinal, combinatorial or high‐throughput chemistry meetings.^60^ Subsequently, instrument vendors such as CEM started their own dedicated conference series on microwave chemistry.^61^ Special symposia were organized at the National ACS Meetings and a variety of other more academically oriented conferences.^62^ In addition, pharma companies were sponsoring meetings on microwave chemistry, either organizing public events or closed door in‐house sessions. In Graz, we started to organize our own *Short Course on Microwave‐assisted Organic Synthesis* (MAOS) series beginning in September of 2003. These two day events proved to be very popular over the years as in addition to formal talks we included lab sessions where the participants (almost exclusively from industry) could see and evaluate microwave reactors from different instrument manufacturers (Figure 9).^63^,^64^

The activities described above essentially meant that during the peak years the microwave chemistry crowd would meet every couple of month (sometimes more often) in different parts of the world to attend a meeting on microwave chemistry.^65^ For academic participants that almost always involved presenting a formal lecture. One can imagine the challenge for speakers trying to include new material in their presentations.^66^

## 5 A Multimillion Euro Grant, Controversy and Scale‐Up

### 5.1 Christian Doppler Laboratory for Microwave Chemistry (CDLMC)

In 2005, in the midst of the golden decade on MAOS, things were running rather smoothly in my laboratory. We were able to develop a number of new interesting synthetic applications and the scientific output and international recognition of our research group in Graz was growing.^67^ However, I felt that there was an urgent need to resolve several key issues in order for microwave chemistry to move forward and to become a generally accepted technology, by both academics, and in particular, by industry. Regardless of the relatively large body of published work on microwave chemistry by 2005, the exact reasons why microwaves would enhance chemical processes were still the subject of some controversy. There was a continuous stream of publications and review articles that appeared in the literature advocating the existence of so‐called “specific or non‐thermal microwave effects”, in addition to the well‐known and rather obvious thermal effects that result from running a reaction at a higher bulk temperature (see Section 5.3).^68^ As someone who, in addition to my training and experience as a synthetic organic chemist, also had a background in physical organic chemistry this was highly unsatisfactory.^69^,^70^ I felt that the question of microwave effects needed to be addressed in a serious manner, given the rapid increase in the use of microwave technology in the chemical sciences at the time, in particular in organic synthesis. Providing a scientific rationale for the observed phenomena appeared even more important, if one considered safety aspects once this technology would move from the small scale laboratory work to pilot or production scale instrumentation. Related to this last issue, the problem on how to scale this technology from gram scale to the kilogram scale and potentially to full production scale also was not resolved in 2005. Our own experience, described in Section 3.3, already made it clear that this was no easy task.

In order to address these challenges I submitted a grant proposal to the Christian Doppler Research Association (CDG) in November of 2005, an Austrian funding agency which promotes the cooperation between universities and industry by supporting application‐oriented basic research in research units termed Christian Doppler Laboratories for up to seven years. Our industrial partners in this grant, officially termed the *Christian Doppler Laboratory for Microwave Chemistry* (CDLMC) initially were Anton Paar and piCHEM, a local company dedicated to peptide synthesis. In subsequent years, ThalesNano (2007), Lonza (2009) and Microinnova (2010) joined the consortium, all interested in the scale‐up aspects of this technology. The substantial amount of funds made available through this grant meant a significant step forward for the group.^71^

### 5.2 Internal Versus External Temperature Measurement and the Monowave 300

One of the first issues that we needed to address was how to properly measure reaction temperature in a microwave‐heated experiment. We were quite pleased with the accuracy of our early investigations back in 2001 using the internal temperature sensor of the MLS multimode system (Figure 3a), that was used quite successfully, for example, for determining reaction kinetics of several solid‐phase organic syntheses.^22^ However, the multimode system proved somewhat impractical for carrying out routine kinetic measurements in series on smaller reaction scale. Our single‐mode systems from Biotage used external infrared (IR) pyrometers that essentially measured the surface temperature of the heavy‐walled glass vessels and not the internal reaction temperature. The physical organic chemist in me always felt a bit unease relying on these measurements, and work published by others had already indicated that the use of these sensors may be unreliable in certain cases.^72^ With the CEM Discover series, while the default way of temperature monitoring for the 10 mL sealed glass reaction vessel also relied on external IR pyrometers, an upgrade was available that made use of a fiber‐optic (FO) probe that could be directly inserted into the reaction mixture, both in open and sealed vessel configuration. This proved very helpful for some initial studies,^73^ however, the software did not allow us to measure and read out both temperature sensors simultaneously. In other words, we could not readily determine if the IR sensor measuring the glass vessel temperature showed something different to the internal FO reaction temperature measurement (which is the one that is really relevant). In a series of investigations that followed, we have therefore used an external FO probe assembly, in some instances using three FO probes positioned at different heights of the reaction vessel simultaneously,^74^ in order to be able to directly compare in real time the results obtained by external IR and internal FO temperature measurement systems. These experiments confirmed that there are indeed many situations where the monitoring of reaction temperature under microwave conditions using external IR pyrometry is unreliable and leads to an erroneous temperature measurement (in extreme cases the internal reaction temperature differed by more than 100 °C from what was measured on the outside).^75^ Since IR pyrometry was the standard method of temperature measurement used in the very popular single‐mode microwave instruments from Biotage and CEM, this meant that many of the stated reaction temperatures given in synthetic microwave papers in those days were most likely inaccurate, including those given in our own publications of course! Our conclusion, derived from many years of research in this area, ultimately was that a simultaneous monitoring and evaluation from the output of both external IR and internal FO probes is the most appropriate and easiest way to measure temperature in microwave‐heated transformations reliably. Ideally, the stirring efficiency of the magnetic stir bar – also of critical importance to the success and reproducibility of a microwave experiment – should additionally be monitored by a built‐in camera.^75^,^76^

**Figure 10:**
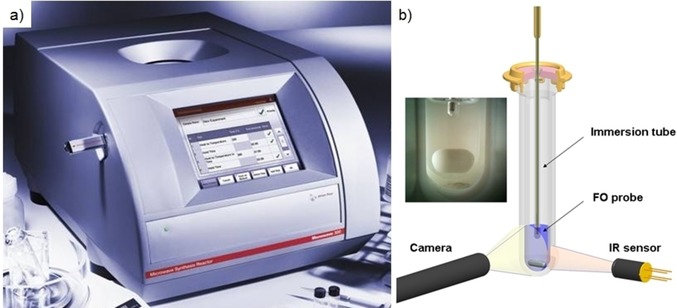
a) Anton Paar Monowave 300 (2009). b) Schematic view of the microwave cavity highlighting dual external (IR) and internal (FO) temperature control and the location of the built‐in camera. The inset shows an image taken by the camera (2 mL liquid volume, magnetic stir bar). Images reproduced with permission from ref. 28b and 76. The operating limits of this instrument were 300 °C and 30 bar pressure.

In the true spirit of the Christian Doppler grant on application‐oriented basic research these findings were eventually reflected in the design and development of the Monowave 300 instrument, a single‐mode microwave reactor launched by Anton Paar in June of 2009 (Figure 10).^77^

### 5.3 Microwave Effects in Organic Synthesis – Myth or Reality?

Having sorted out how to accurately determine reaction temperature in microwave‐assisted transformations we then moved on to investigate the existence of so‐called specific or non‐thermal microwave effects. This topic has always been controversial. Since the first published reports on the use of microwave irradiation to “accelerate” organic chemical transformations dating back to 1986,^8^ there has been considerable speculation and controversy on the exact reasons why microwave irradiation is able to enhance – or otherwise influence – chemical reactions. Much of the debate has centered around the issue if the observed effects can in all instances be rationalized by purely thermal/kinetic phenomena (thermal microwave effects), as a consequence of the rapid heating and high bulk reaction temperatures that can be attained using microwave heating (especially in sealed vessels), or whether some effects are indeed associated to a selective interaction of the electromagnetic field with specific molecular entities in the reaction mixture *not* connected to a macroscopic bulk temperature effect (so‐called specific or nonthermal microwave effects).^7^,^28^,^68^ The latter two types of microwave effects have been proposed when the outcome of a chemical transformation performed under microwave conditions was significantly different from the conventionally heated counterpart *at the same measured bulk reaction temperature*.

In 2001, *Angewandte Chemie* published a short highlight article by Nikolai Kuhnert entitled “Microwave‐assisted reactions in organic synthesis‐are there any nonthermal microwave effects?”.^78^ The generalized conclusion of the author, referring mainly to the specific examples we published on the Biginelli reaction^19^ and on solid‐phase synthesis,^22^ was: “definitely not is the answer!”^78^ This publication resulted in a rather angry email of several members of the French microwave chemistry community to the Editor of *Angewandte Chemie*, as the conclusion presented in this article sharply contradicted what had been presented in a review on microwave effects published the same year.^7^ In formulating the CDLMC grant proposal on this topic I thought, that from a scientific standpoint of view, it would be most logical to initially repeat some of the experiments others had published where specific or non‐thermal microwave effects had been found. This way we could learn very quickly if such effects in fact existed or, if not, what the problem with the original experimental design or interpretation of results was. To make a very long story – that has already been told elsewhere^79^,^80^ – very short: in all the comparison experiments between microwave heating and conventional heating *applying the exact same adequately measured reaction temperature profiles* we have never found any effect that ultimately could not be rationalized by a purely bulk temperature phenomenon.^81^ These general findings were not restricted to traditional organic synthesis, but also held true for solid‐phase peptide synthesis, enzymatic resolutions, peptide hydrolysis, proteolytic digestion, inorganic nanomaterials/polymer synthesis and microwave‐assisted extractions.^82^ In each and every case, when carefully conducted control experiments were performed – paying meticulous attention to all relevant process parameters – even the most spectacular rate enhancements, changes in selectivity or material properties could be duplicated by conventional heating.^83^

**Figure 11:**
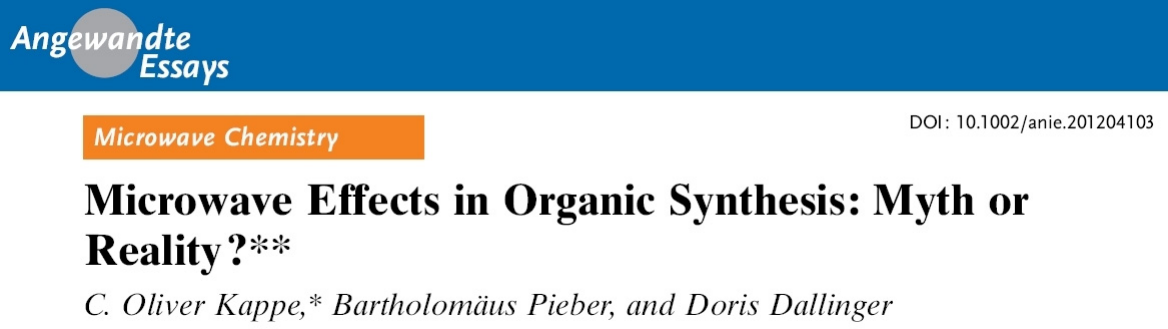
MythBusters in action: The title of our Essay in *Angewandte Chemie* in 2013 (ref. 79).

What I had underestimated at the time was the fact that I would probably not be making a lot of friends by repeating experiments other scientists had published and arrive at different conclusions or find errors in their experimental design. A well‐publicized case was our debate with the Dudley group at Florida State University in 2013. In short, we had repeated microwave experiments performed by Dudley where effects had been proposed that could not be explained by purely bulk temperature phenomena.^84^ These results had been heralded by an RSC journal as the revival of the magic microwave effects debate (choosing a scientifically rather inappropriate title),^85^ whereas the general consensus in the community at that time was that this controversy had essentially been settled.^86^ Our results published in 2013 as part of an Essay in *Angewandte Chemie* on microwave effects strongly indicated that erroneous temperature measurement was involved in the experiments carried out by the Dudley group (Figure 11).^79^,^87^

In addition to the disagreement between academic groups on the existence/non‐existence of various types of microwave effects, there was an economically much more relevant clash going on behind the scenes among the manufacturers of microwave equipment. It was rather obvious to most experts in the field that the use of certain technologies and processing conditions promoted by specific instrument vendors would only make sense if the user would believe that microwave chemistry is more than a heating technique. For example, if one accepts the proviso that specific/non‐thermal microwave effects do not exist, then to operate a microwave reactor under reflux conditions at the boiling point of the reaction mixture does not appear to be a particularly useful method, since the results obtained under these conditions can typically be duplicated easily by conventional conductive heating using heating mantles or oil baths.^88^

As the seven year funding period of the CDLMC was coming to an end in June of 2013,^71^ and I felt we had resolved all open questions relating to temperature measurement and the existence of specific or non‐thermal microwave effects, our main conclusions with respect to these matters were written up in three review‐style articles that were published in 2013.^75^,^79^,^80^ In following the literature published since that time, I have not come across anything that convinces me that specific or non‐thermal effects exist; I rather suspect that erroneous temperature measurement is at play in those cases where such effects have been claimed.^89^

### 5.4 From Grams to Kilograms and Going Parallel and Small Again

With few exceptions most examples of microwave‐assisted synthesis published before 2005 were performed on a less than 1 g scale (typically 1–5 mL reaction volume). This was in part a consequence of the availability of single‐mode microwave technology that allowed the safe processing of small reaction volumes under sealed vessel conditions by microwave irradiation. It appeared to me that for microwave‐assisted synthesis to be become a fully accepted technology in the future there was a need to develop larger scale techniques, that could ultimately routinely provide products on a multi kg (or even higher) scale. At that time, two different philosophies for microwave synthesis on larger scale (>100 mL processing volume) had emerged. While some groups have employed larger batch‐type multimode reactors (often using rotor systems containing a number of individual vessels), others have used continuous flow techniques using single‐ and multimode instruments to overcome the inherent problems associated with microwave chemistry scale‐up.^28^ We had experimented with both technologies in Graz. As a follow‐up to the work described in Section 3.3 using multi‐vessel rotor systems in multimode instruments – and based on significant industrial demand – Anton Paar subsequently developed an instrument having a single 1 L PTFE vessel that could be operated at up to 250 °C and 30 bar pressure using a mechanical overhead stirring system.^90^,^91^ Although even larger batch microwave systems for closed vessel operation have been designed, we felt that owing to penetration depth issues and safety concerns this was probably the limit where one could and should go with batch microwave technology.

In 2007, ThalesNano joined the CDLMC, followed by Lonza in 2009 and Microinnova in 2010. All three companies had a strong interest in continuous flow chemistry, ThalesNano from the standpoint of developing reactors for labscale use, Lonza and Microinnova in the context of large‐scale manufacturing. The fast reactions possible under sealed vessel microwave batch conditions appeared extremely attractive from the manufacturing perspective, leading to potentially very high throughputs in properly designed high‐temperature flow reactors. The question that needed to be addressed first was: is there any benefit from using microwave heating for flow chemistry applications? We had experimented with our two CEM flow microwave reactors for some time,^92^ but could not see an immediate benefit for lab‐scale operations using these devices, in particular as we already had encouraging experiences with flow chemistry using conventionally heated devices from 2004 onwards.^44^,^45^,^93^ Our collaboration with all three companies in the framework of the CDLMC for the remaining years of the grant therefore focused on the question on how to translate the results obtained in small‐scale sealed vessel batch microwave systems to scalable and safe high‐temperature/high‐pressure continuous flow conditions and will therefore not be further discussed herein.^94^

At the same time, as we went up in scale we also thought about miniaturization and parallelization, again a leftover of my longstanding interest in combinatorial chemistry. As outlined above using single‐mode processing aided by robotics proved extremely valuable for synthetic chemists both in academia and industry (*cf*. Figure 8). However, it appeared to us that the automated sequential processing strategy could become impractical when a large number of optimization experiments need to be performed, as for example in the context of a statistical “Design of Experiment” (DoE) campaign, or in the context of a large library synthesis project (>100 compounds). In those instances, the time‐saving aspect associated with microwave chemistry may be compromised by having to irradiate each reaction vial individually, and the utilization of a parallel microwave processing technique would clearly be advantageous. In 2007, we had shown that using multi‐vessel rotor systems in a multimode instrument (*cf*. Figure 5) compound libraries could be prepared in a single irradiation event by translating optimized conditions from single‐mode experiments to a 48‐vessel rotor system (filling volume of 6.0–25 mL per vessel).^95^ Using the Liebeskind‐Srogl chemistry outlined in Scheme 1h as an example, a subset of a 30 member library of 5‐aroyl‐3,4‐dihydropyrimidin‐2‐ones was made in one single microwave irradiation event that lasted 1 hour. In order to prepare a 30 member library under those conditions using automated sequential processing a time span of 30 h would be needed.^95^

**Figure 12:**
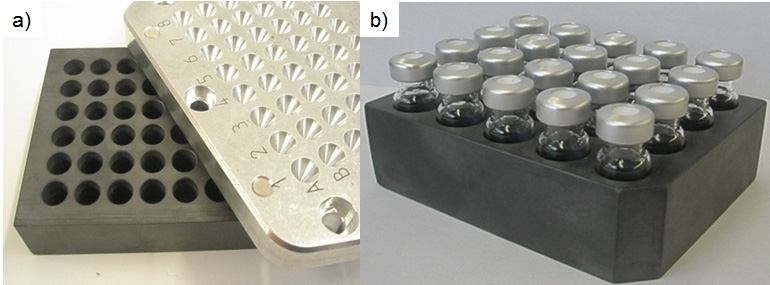
Silicon Carbide plate formats for high‐throughput experimentation (Anton Paar, 2007 and 2009). a) SiC Plate (8×6 matrix): reactions are performed directly inside the bore holes of the SiC block. b) SiC Plate (5×4 matrix): reactions are performed in standard HPLC/GC autosampler vials fitted with aluminum crimp tops. The set‐up can additionally be equipped with a sealing top plate (not shown). Reproduced with permission from ref. 98.

**Figure 13:**
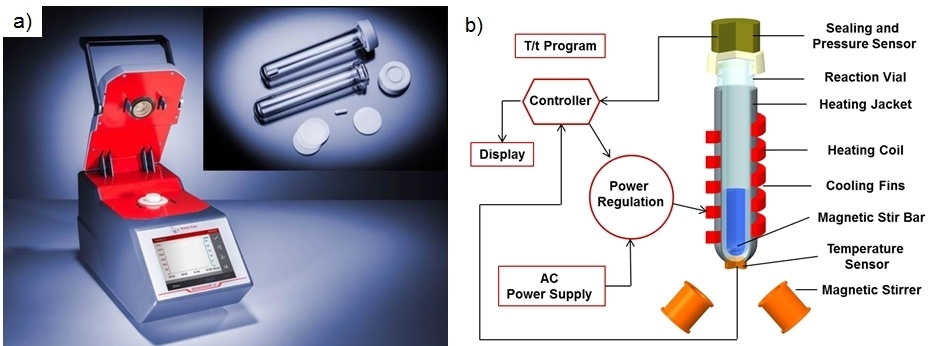
a) Anton Paar Monowave 50 with open cover and 10 mL glass vials (2016). b) Schematic drawing depicting the concept of a conductively heated sealed vial reactor employing a 10 mL glass vial. Reproduced with permission from ref. 102.

Driving this concept further, we subsequently developed microtiter plate systems made out of strongly microwave‐absorbing silicon carbide (SiC) ceramic material. Similar to the use of this material in a single‐mode microwave device,^80^ its use under multimode conditions and in microtiter plate format makes it irrelevant what is contained inside the wells of the plate, as the SiC plate itself is heated by microwaves, not the contents inside the plate.^96^ Since the semiconducting plate material possesses high thermal conductivity, no temperature gradients across the microtiter plate exist. Therefore, many of the disadvantages experienced in attempting to perform microtiter plate chemistry applying standard microwave conditions could be eliminated.^97^ The SiC‐based microtiter platforms allowed sealed vessel processing of small reaction volumes at a maximum temperature/pressure limit of 200 °C*/*20 bar (Figure 12).^98^ Notably, a platform type utilizing HPLC/GC vials as reaction vessels (Figure 13b) allowed analysis directly from the reaction vessel eliminating the need for a transfer step from the reaction to the analysis vial.^98^ The latter system has been used extensively by our group not only for library synthesis and reaction optimization/screening, but also in the context of several (bio)analytical applications.^98^

### 5.5 Post Scriptum: A Microwave that's not a Microwave – The Monowave 50

After having performed a shear endless number of comparison experiments between microwave and conventionally heated reactions over the years,^82^ it was clear to us that microwave chemistry should probably best be defined as: “an incredibly effective, safe, rapid, and highly reproducible way to perform an autoclave experiment under strictly controlled processing conditions”.^79^ In my 2013 *Account of Chemical Research* article, I further posed the question:^80^ “what really is the difference between a microwave‐heated reaction and an autoclave experiment?” The answer I provided was: convenience! Using state‐of‐the art single‐mode microwave technology, reaction mixtures can be rapidly superheated far above their boiling points in an operationally simple and safe manner with exquisite online control over reaction temperature and pressure. All this is very hard to do using currently available conventional autoclaves or sealed vessel reaction systems. The question became: can we design a sealed vessel autoclave system that makes use of all the nice features we like about our microwaves but does not have a magnetron?

In 2009, we had introduced a 10 mL reaction vessel made out of silicon carbide (SiC) for use in single‐mode microwave reactors.^99^ This was done for purely academic reasons in order to be able to efficiently mimic a conventionally heated autoclave experiment.^80^ Notably, microwave irradiation will induce a flow of electrons in the semiconducting SiC ceramic that heats the material very efficiently through resistance (ohmic) heating mechanisms. Owing to its extremely high thermal conductivity and effusivity (a measure for the ability to exchange thermal energy with its surroundings) the heat flow through the wall of the SiC reaction vessel is exceptionally fast in both directions. The contents of the reaction vessel are therefore also heated very rapidly, regardless of their microwave absorptivity.^80^,^99^ It occurred to one of my PhD students, David Obermayer, that ohmic heating of the SiC reaction vessel should, as an alternative to microwave irradiation, be much easier to achieve by simply applying surface electrodes on the material. A first prototype of a resistance‐heated SiC autoclave of this nature featuring on‐line temperature and pressure monitoring/control, magnetic stirring, and being capable of operating at temperatures of up to 250 °C and pressures up to 24 bar was assembled during 2012/2013.^100^ It became apparent rather quickly that this device was, in principle, able to mimic what could be achieved with state‐of‐the‐art single‐mode microwave technology as several control experiments demonstrated.^101^

The basic concept was taken up by Anton Paar and led to the development of the Monowave 50 reactor (Figure 13), an instrument that enables sealed vessel heating of 10 mL tubes to 250 °C and 20 bar pressure by applying conductive heating principles.^102^ It employed the same glass vessels^103^ as the Monowave 300^104^ microwave reactor *(cf*. Figure 10) and many of the design features of this instrument were implemented into this autoclave‐type reactor.^102^ Similar to the prototype instrument it essentially mimics what can be achieved in a standard single‐mode microwave reactor.^102^ Given its small footprint and low‐cost, we have recently implemented the Monowave 50 into our undergraduate organic chemistry teaching labs at the University of Graz.^105^

I would argue that the development of the Monowave 50 is another nice example that demonstrates how an idea originally conceived and developed purely out of academic interest^99^ can subsequently lead to an invention,^100^ and ultimately to a commercial product (Figure 13). Rather appropriately, this was the last major project in the group related to our CDLMC grant.

In addition to the work that was going on in the CDLMC described in this section a significant number of other MAOS projects was pursued in the group at the same time,^106^ in many instances involving international collaborations and visiting students or postdoc (for example, with Rodrigo O. M. A. de Souza in Rio de Janeiro, Brazil, and Rafa Luque in Cordoba, Spain).^107^

## 6 The End of a Golden Era

**Figure 14:**
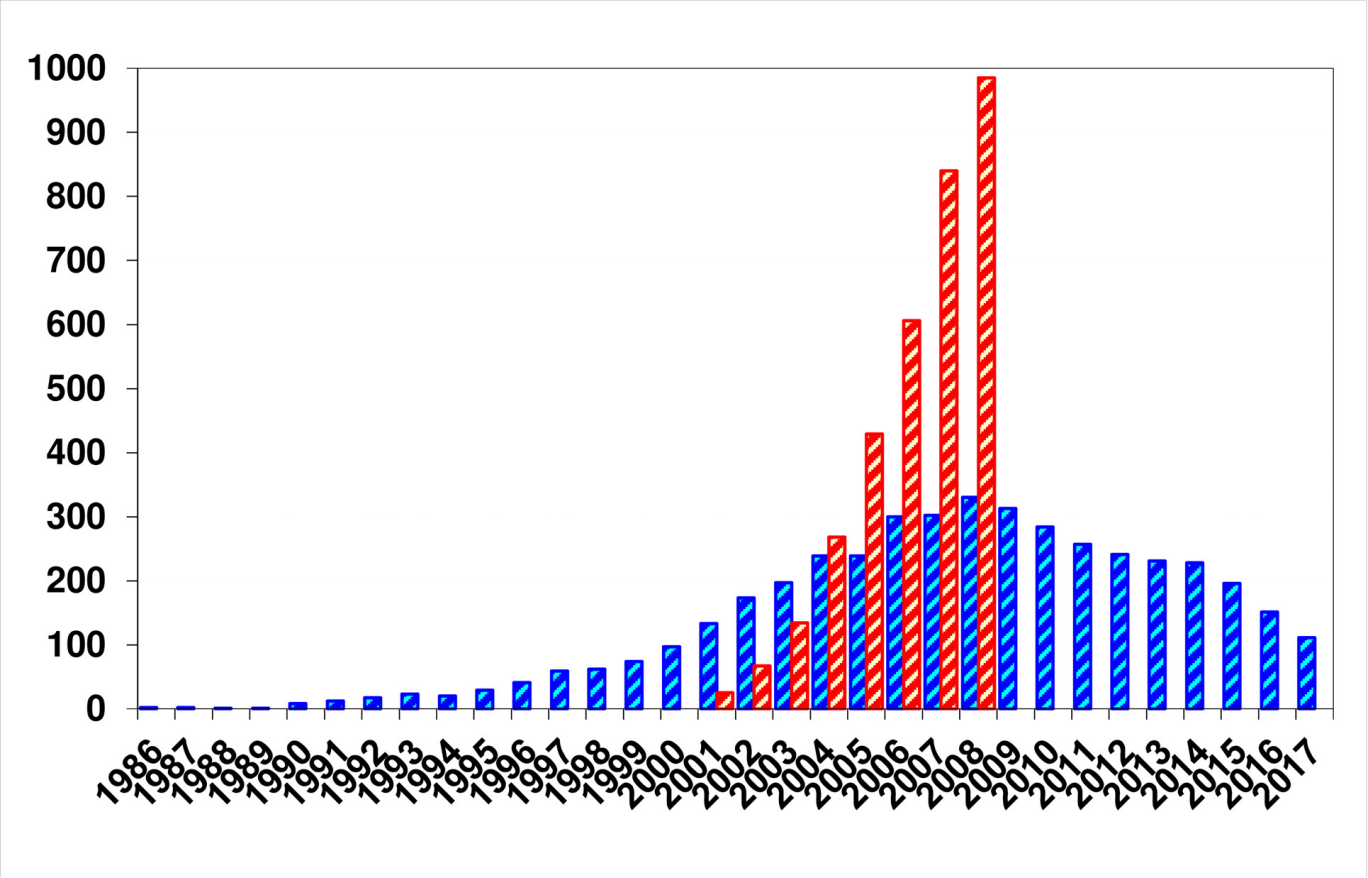
Publications on microwave‐assisted organic synthesis (1986–2017). Blue graphs: Number of articles involving MAOS for seven selected synthetic organic chemistry journals (*J. Org. Chem*., *Org. Lett*., *Tetrahedron*, *Tetrahedron Lett*., *Synth. Commun*., *Synthesis*, *Synlett*. SciFinder Scholar search, keyword: “microwave”). The red graphs represent the number of publications (2001–2008) reporting MAOS experiments in dedicated reactors with adequate process control (ca. 50 journals, full text search: microwave).

At the end of the first decade of this century it was clear that the heydays of microwave‐assisted organic synthesis were over. The number of conferences and short courses related to microwave chemistry sharply decreased after 2009, at the same time we also noticed – much to my surprise I must admit – that the number of publications started to decrease (Figure 14). As outlined in Section 4, we had stopped performing full text searchers in journals in 2008, but subsequently found that in order to get a rough idea on activities in MAOS we could readily perform a keyword search (“microwave”) in SciFinder and then refine the results to journals that almost exclusively publish research in the field of synthetic organic chemistry (Figure 14). Although one must be careful not to put too much significance into these metrics,^108^ it is indisputable that the number of publications that focus on MAOS has decreased significantly since 2008.

Aligning with Gartner's hype cycle this was also a “period of disillusionment”, and there were several instances where microwave technology had failed to deliver and instruments (and sometimes companies) disappeared from the market. Browsing through the relevant book chapters and reviews on microwave equipment,^28^ one cannot help noticing that many pieces of microwave hardware that were launched during the heydays do not exist anymore today, or are irrelevant in terms of market share. Apart from the venerable Smith Synthesizer of 2000 and other highly automated and perhaps over‐engineered robotic platforms that followed, this is particularly true for hyphenated techniques (i. e., microwave instruments coupled with flow reactors, ultrasound or in‐line analysis), hybrid single/multimode devices, and, most notably, batch scale‐up systems that go beyond the 1 liter scale.

So the question I ask myself is: has MAOS failed? In the interview with *Nature* in 2003 I had stated: “In 10–15 years, we will see a microwave reactor in every academic and industrial laboratory.”^55^ This was probably a bit too optimistic and certainly has not become reality, at least not as far as the academic world is concerned. The prediction made in the Evalueserve study of 2005 that the sales of synthesis equipment will overtake the ones for digestion has also not materialized.^50^ Although it is hard to get exact figures from instrument vendors, it is fair to say that none of them is overly excited about the number of microwave reactors for synthesis they are currently selling, and more business is still made with digestion units. One reason given early on was the high price tag of first generation single‐mode devices. Prices have now come down to way below 10.000 € for basic units so it is difficult to still use this argument. Another more general viewpoint pertains to a perceived resistance of the (often viewed as being rather conservative) organic chemistry community to move away from round‐bottom flasks and heating mantles to something different. I believe there is some truth to that. Perhaps it has also something to do with the fact that microwave‐assisted organic synthesis has never moved beyond the kilogram scale and there are no documented cases for its use in large scale batch chemical manufacturing.^109^

Whatever the reasons, while I would not say that microwave chemistry has failed, the acceptance and penetration of this technology throughout the scientific community has been lower than expected. Although for discovery‐type operations in the pharmaceutical industry microwave chemistry has been fully embraced (*cf*. Figure 8),^110^ this is evidently not the case in most academic labs. In browsing through recent publications on synthetic organic chemistry I often cannot help thinking that a significant fraction of these transformations would probably run much more efficiently under microwave conditions (and I am not only referring to reaction rates). Looking at the publication metrics given in Figure 14, and putting those data into context with the overall number of publications in organic synthesis, it becomes clear that only a very small fraction of synthetic organic chemists is using microwave technology today (irrespective of the fact if their lab owns a microwave or not).^111^,^112^ The number of research groups in organic chemistry that focus their activities on microwave chemistry and/or technology today is probably in the single digit range, most likely since there is no funding for doing so.^113^ Notably, some of the pioneers in the field have retired, others have moved on to different areas (like my group has). So in returning to Gartner's hype cycle one last time: I think we have now reached the “plateau of productivity”, and although on a significantly lower level than perhaps initially expected, microwave chemistry has today received mainstream adoption. Certainly in our laboratory we utilize single‐mode microwave reactors as a tool on an almost daily basis as we hardly use round bottom flask anymore to heat reaction mixtures on small scale. So in a way microwave reactors have become, at least for us, “The Bunsen burner of the 21^st^ century.”^114^

## 7 Concluding Remarks

My involvement in the field of microwave chemistry for the past two decades has been an incredibly exciting and rewarding scientific journey. I have very much enjoyed working on the interface of purely academic and curiosity‐driven research on the one hand, and at the same time being able to address topics more related to applied research and instrument development.^114^ Like most scientists, I experience a distinct feeling of satisfaction in knowing that the results of our research are not only appreciated but also used in practice by others in the field.

By expanding our research related to microwave chemistry beyond my original field of expertise and training (organic synthesis), I was forced to acquaint myself with scientific areas that were very new to me. Starting this journey in 1998, I could have never predicted that years later I would publish scientific articles on synthesizing inorganic nanocrystals, polymer synthesis, proteolytic digests, and on the extraction of caffeine from Nespresso capsules (in the context of forensic investigations we have even made heroin in a microwave).^82^ Naturally, much of this work was only made by possible by collaborations with many different scientists, both locally and abroad. Visiting our collaborators and attending countless conferences and workshops around the globe during those years allowed me to see parts of the world that I would otherwise perhaps not have visited.

The question that comes to my mind in looking back at the very beginnings of my involvement in microwave chemistry is: what would have happened if I had not attended the 1998 conference in Eger,^4^ or if Raj Varma would have not given a talk on microwave chemistry at this meeting (*cf*. Section 2.1.)? Would I have still started a career in microwave chemistry? Or would I have turned to something different? Obviously, I will never know the answer to this question but I do know that I have very much enjoyed “my twenty years in microwave chemistry”.

## Biographical Information

*C. Oliver Kappe is Professor of Chemistry at the University of Graz (Austria) and Scientific Director of the Center for Continuous Flow Synthesis and Processing (CC FLOW) at the Research Center Pharmaceutical Engineering GmbH (RCPE). He received his doctoral degree in organic chemistry from the University of Graz.. After periods of postdoctoral research work on reactive intermediates and matrix isolation spectroscopy with Curt Wentrup at the University of Queensland in Brisbane*, *Australia (1993–1994) and on synthetic methodology/alkaloid synthesis with Albert Padwa at Emory University in Atlanta*, *USA (1994–1996)*, *he moved back to the University of Graz in 1996 to start his independent academic career. After a 20 year career in microwave chemistry his current research interests involve continuous‐flow chemistry*, *API manufacturing*, *and process intensification technologies. Since 2011 he has been Editor‐in‐Chief of the Journal of Flow Chemistry*.

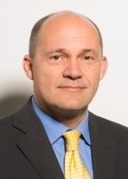

## Supporting information

As a service to our authors and readers, this journal provides supporting information supplied by the authors. Such materials are peer reviewed and may be re‐organized for online delivery, but are not copy‐edited or typeset. Technical support issues arising from supporting information (other than missing files) should be addressed to the authors.

Supplementary

## References

[1] A. Padwa , J. Org. Chem. 2009, 74, 6421–6441. A must read for all young faculty members and I am pretty sure the only paper published in *J. Org. Chem*. that features a Table listing the mountains the author has climbed!19663447

[2] The “Habilitation” defines the qualification to conduct independent research and self-contained University teaching, and is the key for access to a professorship in many European countries. During my Habilitation at the University of Graz (1996–1999) I was funded by a fellowship from the Austrian Academy of Sciences (APART fellowship) which meant I could focus on research and had no formal teaching obligations. I joined the faculty as Assistant Professor in November of 1999 and was subsequently appointed Associate Professor (with tenure) at the University of Graz in March of 2000.

[3] The early part of my research in this area describing improved synthetic methods, mechanistic and conformational studies on dihydropyrimidines was included in the “Habilitation Thesis” of 1998 and was subsequently published in expanded form in: C. O. Kappe , Acc. Chem. Res. 2000, 33, 879–888.11123887

[4] 7th Blue Danube Symposium on Heterocyclic Chemistry, June 7–10, 1998, Eger, Hungary.

[5] Much of the early research work on this topic by the Varma's group is summarized in: R. S. Varma , Green Chem. 1999, 1, 43–55.

[6] A notable exception was an early single-mode reactor design by the French company Prolabo (Synthwave), mainly used by the French microwave chemistry community. A description of this instrument and synthetic applications are summarized in this review article: A. Loupy , A. Petit , J. Hamelin , F. Texier-Boullet , P. Jacquault , D. Mathé , Synthesis 1998, 1213–1234. Around the same time, Strauss developed a dedicated multimode reactor with temperature and pressure control for sealed vessel operation (see ref. 9) which, however, had not been commercialized at that time.

[7] For an early review summarizing several hypotheses related to the existence of so-called specific or non-thermal microwave effects, see: L. Perreux , A. Loupy , Tetrahedron 2001, 57, 9199–9233.

[8a] R. Gedye , F. Smith , K. Westaway , H. Ali , L. Baldisera , L. Laberge , J. Rousell , Tetrahedron Lett. 1986, 27, 279–281;

[9a] C. R. Strauss , R. W. Trainor , Aust. J. Chem. 1995, 48, 1665–1692;

[9b] C. R. Strauss , Aust. J. Chem. 1999, 52, 83–96.

[10] F. Langa , P. de la Cruz , A. de la Hoz , A. Díaz-Ortiz , E. Díez-Barra , Contemp. Org. Synth. 1997, 4, 373–386.

[11a] A. K. Bose , B. K. Banik , N. Lavlinskaia , M. Jayaraman , M. S. Manhas , Chem. Tech. 1997, 27, 18–24;

[11b] A. K. Bose , M. S. Manhas , S. N. Ganguly , A. H. Sharma , B. K. Banik , Synthesis 2002, 1578–1591.

[12] In fact, we had started to experiment with kitchen microwaves and the Biginelli reaction using standard solvents already in late 1997 or early 1998. At that time a master degree student (Fabio S. Falsone) evaluated the use of a sealed PTFE autoclave in a standard kitchen microwave. Similar to the experience Gedye made already in 1986 (ref. 8a) this resulted in a “violent explosion”. In our case, a rupture of the pressure safety disk in the PTFE autoclave prevented a major disaster, but even so a massive cleaning operation of the microwave oven from the leaked reaction mixture was required. This line of experiments was stopped soon thereafter in frustration and no results were ever published.

[13] C. O. Kappe , F. S. Falsone , Synlett 1998, 718–720.

[14] This is the experimental as it appears in the *Synthesis* publication: “The appropriate ß-keto ester (2.2 mmol), aldehyde (2.0 mmol), urea derivative (6.0 mmol) and PPE (300 mg) was placed (in that order) in a 20 mL glass beaker. After the mixture had been stirred for 10–20 s with a spatula the reaction container was inserted into a 400 mL Pyrex beaker filled with neutral alumina (150 g, 100–290 mesh). This set-up was irradiated in the MW oven 3 times at the 50 % power level for 30 s (25 % power level for entries **4 l** and **4 o**, 5 times 50 % power level for entry 4k) with a 1 min and 2 min cooling period after the 1st and 2nd irradiation cycle, respectively.” As most people would agree today, this kind of experimental is asking for trouble in terms of reproducibility, considering the uneven field homogeneities and different power outputs of the various kitchen microwaves on the market. Also note that there is no reaction temperature given.

[15] C. O. Kappe , D. Kumar , R. S. Varma , Synthesis 1999, 1799–1803.

[16] Jubiläumsfonds der Oesterreichischen Nationalbank, Project 7904: “Chemoenzymatic Synthesis of Enantiomerically Pure Dihydropyrimidones” (1999–2000). In re-reading the 1999 grant application I now realize that we originally had planned to purchase a single-mode system from Prolabo (see ref. 6). With the limited funds available from this grant I had to opt for the MLS multimode system. In addition to the planned microwave work outlined in the proposal (not reflected in the project title) we did also manage to do chemoenzymatic syntheses. For details, see: B. Schnell , W. Krenn , K. Faber , C. O. Kappe , J. Chem. Soc. Perkin Trans. 1, 2000, 4382–4389.

[17] MLS GmbH is internationally better known via its worldwide distributor Milestone s.r.l. in Italy.

[18] Austrian Science Fund (FWF) Project P-11994-CHE: “Synthesis of Biologically Active Dihydropyrimidine Derivatives” (January 1997–September 2001). The originally approved 36 month funding period of this grant was subsequently extended by 21 months on a cost neutral basis, which helped significantly to fund my early research activities on microwave chemistry, in particular providing funding for my first PhD student. In hindsight, it was perhaps a blessing that I was not able to recruit a PhD student in the first 36 months, otherwise the funds would have been used up by 1999 as original planned.

[19] A. Stadler , C. O. Kappe , J. Chem. Soc. Perkin Trans. 2, 2000, 1363–1368.

[20] *Microwave-Enhanced Chemistry. Fundamentals*, *Sample Preparation and Applications*, (Eds. H. M. Kingston, S. J. Haswell), American Chemical Society, Washington D. C. **1997**. Note that in this 1997 book of 750 pages there was only one ca. 50 page chapter on MAOS.

[21a] R. Gupta , A. K. Gupta , S. Paul , P. L. Kachroo , Ind. J. Chem. 1995, 34B, 151–152;

[21b] A. Dandia , M. Saha , H. Taneja , J. Fluorine Chem. 1998, 90, 17–21.

[22] A. Stadler , C. O. Kappe , Eur. J. Org. Chem. 2001, 919–925. Similar to our work on the Biginelli reaction described in ref. 19 we have performed careful comparison experiments between microwave and conventional heating for reactions on solid-phase using internal temperature measurements, but could not find any difference in results between the two heating methods.

[23a] A. Stadler , C. O. Kappe , Tetrahedron 2001, 57, 3915–3920.

[23b] G. A. Strohmeier , C. O. Kappe , J. Comb. Chem. 2002, 4, 154–161; 11886290 10.1021/cc010043r

[23c] A. Stadler , S. Pichler , G. Horeis , C. O. Kappe , Tetrahedron 2002, 58, 3177–3183.

[24] T. Razzaq , C. O. Kappe , Tetrahedron Lett. 2007, 48, 2513–2517.

[25] M. Damm , C. O. Kappe , Mol. Diversity 2009, 13, 529–543. 10.1007/s11030-009-9167-3 19548098

[26] For a representative key example from this time period, see: N.-F. K. Kaiser , U. Bremberg , M. Larhed , C. Moberg , A. Hallberg , Angew. Chem. Int. Ed. 2000, 39, 3596–3598. 10.1002/1521-3773(20001016)39:20<3595::aid-anie3595>3.0.co;2-s 11091409

[27] In simple terms: While a modern laboratory multimode instrument, in principle, has the same basic design concept as a kitchen microwave, in a single-mode unit the reaction vessel is placed directly in the wave guide at a location of high field intensity. For details, see ref. 28.

[28] For more detailed information on all aspects of microwave chemistry including equipment technology, scale-up and microwave effects the reader is referred to the two most recent comprehensive books available on the subject:

[28a] *Microwaves in Organic Synthesis*, 3rd ed. (Eds. A. de la Hoz, A. Loupy), Wiley-VCH: Weinheim, **2013**.

[28b] C. O. Kappe , A. Stadler , D. Dallinger , Microwaves in Organic and Medicinal Chemistry , 2nd ed., Wiley-VCH, Weinheim, 2012.

[29] The instrument was originally designed to speed up the preparation of radiopharmaceuticals: For details, see: S. A. Stone-Elander , N. Elander , J.-O. Thorell , G. Solås , J. Svennebrink , J. Labelled Compd. Radiopharm. 1994, 10, 949–960.

[30] First Conference on Coherent Synthesis, April 12–14, 2000, Cambridge, UK. The 750 USD registration fee seemed enormous to me at the time but considering the events that followed the money was well spent.

[31] For a detailed description of the Coherent Synthesis concept, see:

[31a] J.-S. Schanche , Mol. Diversity 2003, 7, 293–300. An official price tag of 190.000 USD for the Smith Synthesizer was published in a 2002 report:

[31b] *Instrumenta* **2002**, *18*(22), 6.

[32a] A. Stadler , C. O. Kappe , J. Comb. Chem. 2001, 3, 624–630. A more detailed procedure was subsequently published:11703160 10.1021/cc010044j

[32b] D. Dallinger , C. O. Kappe , Nat. Protoc. 2007, 2, 1713–1721. 17641636 10.1038/nprot.2007.224

[33] The Smith Synthesizer was later renamed Emrys Synthesizer/Liberator and was subsequently replaced by different models with a lesser degree of automation (e. g., Emrys Optimizer and Creator launched in 2001). In September of 2003 Personal Chemistry was acquired by Biotage and the current instrument model used in most academic and industrial laboratories, including ours, is the Biotage Initiator^+^. Support by Biotage for our activities in the microwave field in a variety of different formats remained in place for many years. The concept of Coherent Synthesis faded away over time but given the current interest of the synthetic organic community in robotics, automation, self-optimizing reactors and the use of artificial intelligence and machine learning in synthesis, I can't help thinking that the Smith Synthesizer and the Coherent Synthesis concept developed in the late 1990’s already had incorporated some of these features that we get so excited about today. See, for example:

[33a] D. T. Ahneman , J. G. Estrada , S. Lin , S. D. Dreher , A. G. Doyle , Science 2018, DOI: 10.1126/science.aar5169.

[33b] D. Perera , J. W. Tucker , S. Brahmbhatt , C. J. Helal , A. Chong , W. Farrell , P. Richardson , N. W. Sach , Science 2018, 359, 429–434. 29371464 10.1126/science.aap9112

[34] Unrelated to our activities in microwave chemistry at the time I started a collaboration with BASF in 2000 that provided funding for my 2^nd^ PhD student, Gernot A. Strohmeier, to work on the synthesis of 1,3-thiazines libraries involving both polymer-supported reagents and solid-phase synthesis. See, for example: G. A. Strohmeier , C. O. Kappe , Angew. Chem. Int. Ed. 2004, 43, 621–624; 10.1002/anie.200352731 14743421

[35] This C&EN article reflects the status and enthusiasm about combinatorial chemistry in mid-2001: S. Borman , Chem. Eng. News 2001, 79, 49–58. For an early review on combinatorial synthesis assisted by microwave heating, see:

[36] Austrian Science Fund (FWF) Project P15582-N03: “Microwave-Assisted Combinatorial Chemistry” (October 2002–September 2005). The first submission of a grant with a similar title to a FWF funding scheme was in November of 1999. After four attempts, the grant was finally approved in May of 2002.

[37] I note that our synthetic efforts in library synthesis were rewarded by discovering compounds with biological activity. For details, see:

[37a] H. Prokopcová , D. Dallinger , G. Uray , H. Y. K. Kaan , V. Ulaganathan , F. Kozielski , C. Laggner , C. O. Kappe , ChemMedChem 2010, 5, 1760–1769. 20737530 10.1002/cmdc.201000252

[37b] Y. K. Kaan , V. Ulaganathan , O. Rath , H. Prokopcová , D. Dallinger , C. O. Kappe , F. Kozielski , J. Med. Chem. 2010, 53, 5676–5683 20597485 10.1021/jm100421n

[38] The results obtained during this period (2002–2005) were published in ca. 25 articles and have been summarized in: C. O. Kappe , A. Stadler , D. Dallinger , G. Strohmeier , R. Perez , O. I. Zbruyev , N. Stiasni , P. Walla , N. Gorobets , B. Yousefi , N. Mont , B. Desai , A. Lengar , K. Krascsenicsová , S. Garbacia , B. Khanetskyy , T. N. Glasnov , J. M. Kremsner , A. Gomez da Silva , B. Basca , in “ New Methodologies and Techniques for a Sustainable Organic Chemistry”, A. Mordini, F. Faigl (Eds.), Springer, Dordrecht, 2008, pp 225–251. The long list of authors reflects the contributions from many different individuals working in my labs at the time.

[39] For a representative example involving 5 consecutive microwave steps, see: T. N. Glasnov , W. Stadlbauer , C. O. Kappe , J. Org. Chem. 2005, 70, 3864–3870. 15876072 10.1021/jo0502549

[40] A. Stadler , B. H. Yousefi , D. Dallinger , P. Walla , E. Van der Eycken , N. Kaval , C. O. Kappe , Org. Process Res. Dev. 2003, 7, 707–716.

[41] N. Kaval , W. Dehaen , C. O. Kappe , E. Van der Eycken , Org. Biomol. Chem. 2004, 2, 154–156. 14737635 10.1039/b315150f

[42] The Synthos 3000 was subsequently developed into an instrument called Multiwave PRO that now serves both the digestion and synthesis market. It was the first of several microwave synthesis systems developed by Anton Paar over the years (see below) and we have continued to collaborate with Anton Paar ever since that time.

[43] J. M. Kremsner , C. O. Kappe , Eur. J. Org. Chem. 2005, 3672–3679.

[44] In a collaborative project with the groups of Andreas Kirschning and Ulrich Kunz in Germany (Austrian Science Fund Project I18-N07, Microwave-accelerated catalysis in flowthrough reactors, December 2003–November 2007) we have studied the characteristics of several Pd-catalyzed transformations (using immobilized Pd catalysts) under batch and flow microwave conditions. For details, see: K. Mennecke , R. Cecilia , T. N. Glasnov , S. Gruhl , C. Vogt , A. Feldhoff , M. A. L. Vargas , C. O. Kappe , U. Kunz , A. Kirschning , Adv. Synth. Catal. 2008, 350, 717–730. The funds from this grant allowed the purchase of our first CEM Discover system (Voyager) late in 2003 and provided support for a PhD student in Graz, Toma N. Glasnov.

[45] In 2004, we started a collaboration with ThalesNano (Budapest, Hungary) for evaluating the capabilities of the H-Cube high-temperature/high-pressure flow hydrogenator. This has led to one of the first publications using this device: B. Desai , C. O. Kappe , J. Comb. Chem. 2005, 7, 641–643 and kicked-off our collaboration with ThalesNano that has continued ever since.16153056

[46] M. Larhed , A. Hallberg , Drug Discovery Today, 2001, 6, 406–416. 11301285 10.1016/s1359-6446(01)01735-4

[47] P. Edwards , Drug Discovery Today, 2001, 6, 614. 11408194 10.1016/s1359-6446(01)01846-3

[48] The CEM Discover has undergone several modifications since its launch but remains the base module of all of CEM's single-mode microwave reactors for organic- and peptide synthesis, and various life science applications. An automated version (CEM Explorer) was introduced in 2002. CEM also offers multimode instruments for organic synthesis (MARS 6).

[49] The first publication using the CEM Discover came from Nicholas E. Leadbeater (UK) who subsequently became one of the main players in the field of MAOS: N. E. Leadbeater , H. M. Torenius , J. Org. Chem. 2002, 67, 3145–3148. 11975584 10.1021/jo016297g

[50] Evalueserve 2005 Report “Developments in Microwave Chemistry”: For details, see: http://www.rsc.org/images/evaluserve_tcm18-16758.pdf

[51] I know of one North American academic who was offered a free (!) instrument from company A, if he would decide not to purchase (!) an instrument from company B. He stayed with company B in the end.

[52] At about the same time the first chemistry journals started to effectively ban the publication of results derived from the use of kitchen microwave instruments, citing lack of reproducibility and safety concerns. For the *Journal of Organic Chemistry*, for example, this is still part of the official *Guidelines to Authors*. I was a strong supporter of this move and helped establishing these guidelines in 2004.

[53] The results of this exercise were ultimately summarized in a 123 page long article containing more than 700 synthetic schemes and 900 references: C. O. Kappe , D. Dallinger , Mol. Diversity 2009, 13, 71–193. Up till 2008, selected examples were published online on a monthly basis via the Organic Chemistry Portal: http://www.organic-chemistry.org/Highlights/2008/index.shtm. Since we also noted down which microwave instrument was used in each of the publications we had a pretty good overview on trends in the equipment market. For example, for 2008, single-mode devices from either CEM or Biotage were used in more than 90 % of the published articles.

[54] I am amazed and humbled at the same time that the *Angewandte Chemie* review I authored in 2004 entitled: “Controlled Microwave Heating in Modern Organic Synthesis” is still the most cited review in microwave chemistry (and by far my most cited publication) getting close to the 3000 citations mark in Web of Science: C. O. Kappe , Angew. Chem. Int. Ed. 2004, 43, 6250–6284; 10.1002/anie.200400655 15558676

[55a] D. Adam , Nature 2003, 421, 571–572. 12571563 10.1038/421571a

[55b] V. Marx , Chem. Eng. News 2004, 82, 14–16.

[55c] N. E. Leadbeater , Chem. World, 2004, 1, 38–41.

[55d] R. Van Noorden , Chem. World, 2008, 5, 40–46.

[56] For a review on microwave-assisted solid-phase peptide coupling, see: S. L. Pedersen , A. P. Tofteng , L. Malika , K. J. Jensen , Chem. Soc. Rev. 2011, 41, 1826–1844. 22012213 10.1039/c1cs15214a

[57a] N. E. Leadbeater , M. Marco , Angew. Chem. Int. Ed. 2003, 42, 1407–1409; 10.1002/anie.200390362 12671982

[57b] R. K. Arvela , N. E. Leadbeater , M. S. Sangi , V. A. Williams , P. Granados , R. D. Singer , J. Org. Chem. 2005, 70, 161–168. It has to be pointed out that the authors did not claim that microwave irradiation was responsible for the observed effects in their original report, but many in the microwave community connected the ability to perform Pd-free cross-couplings to the fact that the reaction was performed by microwave irradiation.15624918

[58] For a review summarizing the advantages of microwave technology for the drug discovery industry, see: C. O. Kappe , D. Dalinger , Nature Rev. Drug Discov. 2006, 5, 51–63. 16374514 10.1038/nrd1926

[59] In a collaboration with Daryl R. Sauer at Abbott Labs the facility shown in Figure 8b was used to synthesize and purify a library of 480 dihydropyrimidine C5 amides in a fully automated fashion (*cf* Scheme 1f). For details, see: B. Desai , D. Dallinger , C. O. Kappe , Tetrahedron 2006, 62, 4651–4664, in particular footnote 21.

[60] As an early example, the microwave theme was part of Cambridge Healthtech Institute's *High-Throughput Organic Synthesis* conference series in San Diego from 2001 till 2005. In 2003, I chaired a full day on microwave chemistry with K. Barry Sharpless giving the opening keynote lecture. The microwave topic was also high on the agenda of SelectBio's *MedChem/MedChem India* and *Advances in Synthetic Chemistry* conference series for many years.

[61] As an example, the CEM *Microwaves in Chemistry* conferences run from 2003 till 2008 and were held mostly in Florida during March, with events in London (2007) and Boston (2008). Biotage held so-called user group meetings for their customers in a variety of different locations.

[62] I organized a special symposium on MAOS at the fall ACS 2004 National Meeting in Boston, and together with Ulrich S. Schubert again at the fall ACS 2008 National Meeting in Philadelphia. The German Chemical Society (GDCh) organized microwave chemistry meetings in Düsseldorf in 2005 and 2007 chaired by Helmut Ritter. Microwaves also featured strongly in the RSC's *High-Throughput Medicinal Chemistry* conference series, similar to events organized by The Japan Combinatorial Chemistry Focus Group (JCCFC) in Japan and meetings of the Society for Combinatorial Sciences (SCS) mainly in Europe (Eurocombi meetings). One of the last international meetings specifically focusing on MAOS was organized in France in 2009: MAOPS – Microwave Assisted Organic and Peptide Synthesis, June 4–5, 2009, La Grande Motte, France. Very well remembered are also the 1^st^ South American Workshop on Microwave Irradiation held in Rio de Janeiro, Brazil (February 3–5, 2010) and a microwave chemistry session at Pacifichem in Honolulu, USA (December 15–20, 2010).

[63] At the time we were the only academic laboratory having instruments from all four instrument vendors which made these courses unique. After running the two day standalone courses in Graz annually from 2003 to 2005, the 2006 event was moved to Budapest and attached to an International Conference held at the same time. The conference featured 18 lectures, 60 poster presentations and had more than 160 participants. Special guests of honor were Rajender S. Varma and Christopher R. Strauss, celebrating the 20^th^ anniversary of the seminal publications by Gedye and Giguere jumpstarting the field in 1986 (see ref. 8). Subsequent MAOS meetings were co-organized in a one-day conference style with SelectBio (San Francisco 2007, Frankfurt 2008) and the Society for Combinatorial Sciences (Beijing, 2009).

[64] One of the last meetings on microwave chemistry, I have organized or chaired was a ZING conference on the Caribbean island of Antigua in 2009. However, it was obvious at the time that we needed to add another topic to the conference (i. e., flow chemistry) in order to get a sufficient number of scientists to the meeting: *Zing Microwave and Flow Chemistry Conference*. The Jolly Beach Resort, Antigua, January 28–31, 2009 (see Figure 9).

[65] In addition to attending conferences, I was teaching short courses on microwave chemistry in different formats for the ACS (with Aubrey Mendonca, see Figure 9) and several other organizations.

[66] I was joking once with a German colleague that we could easily give each other's presentations at the conference since we had heard each other's talks so often that same year. It is difficult to give an exact number but I estimate that between 2000 and 2010 I probably have given ca. 300 presentations on microwave chemistry in a variety of different formats.

[67] In 2004, I received the Prous Science Award for New Technologies in Drug Discovery from the European Federation for Medicinal Chemistry (EFMC) for “innovative work on microwave-assisted organic and combinatorial chemistry”.

[68] For an example of a review on microwave effects from that period containing more than 100 references, see: A. De La Hoz , A. Diaz-Ortiz , A. Moreno , Chem. Soc. Rev. 2005, 34, 164–178. See also ref. 7. 15672180 10.1039/b411438h

[69] In 2004 I had organized a European Science Foundation (ESF) Exploratory Workshop on “Microwave Chemistry and Microwave Effects” in Graz. No agreement on the topic of microwave effects could be reached by the 21 international experts that attended.

[70] Before joining Al Padwa's lab at Emory University in 1994, I spent close to 2 years with Curt Wentrup at the University of Queensland (Brisbane, Australia) as a postdoc and visiting PhD student to work on reactive intermediates which were typically generated by flash vacuum pyrolysis and subsequently characterized in low-temperature matrices using a variety of spectroscopic tools. I believe that the experience gathered during this period was extremely useful for me in our work on microwave effects.

[71] Christian Doppler Laboratory for Microwave Chemistry (Christian Doppler Research Association, CDG, July 2006–June 2013). The 2.3 Mio € from this grant supported a substantial number of postdocs and PhD students during the seven year duration of the grant (Doris Dallinger, Jennifer M. Kremsner, Toma N. Glasnov, Markus Damm, David Obermayer, Bernhard Gutmann, Benedikt Reichart, Stephan Hayden, Bartholomäus Pieber) and allowed purchasing two further CEM Discover instruments for peptide synthesis (Discover SP) and continuous flow processing (Voyager SF).

[72a] M. Nüchter , B. Ondruschka , W. Bonrath , A. Gum , Green Chem. 2004, 6, 128–141.

[72b] N. E. Leadbeater , S. J. Pillsbury , E. Shanahan , V. A. Williams , Tetrahedron 2005, 61, 3565–3585.

[73] M. Hosseini , N. Stiasni , V. Barbieri , C. O. Kappe , J. Org. Chem. 2007, 72, 1417–1424. 17288387 10.1021/jo0624187

[74] M. A. Herrero , J. M. Kremsner , C. O. Kappe , J. Org. Chem. 2008, 73, 36–47. 18062704 10.1021/jo7022697

[75] The reader is referred to a comprehensive tutorial review entitled: “How to measure reaction temperature in microwave-heated transformations” where the details of our studies have been summarized: C. O. Kappe , Chem. Soc. Rev. 2013, 42, 4977–4990. 23443140 10.1039/c3cs00010a

[76] S. Hayden , M. Damm , C. O. Kappe , Macromol. Chem. Phys. 2013, 214, 423–434.

[77] For our role in the development of this instrument the Christian Doppler Laboratory for Microwave Chemistry was awarded the 100.000 € Dr. Wolfgang Houska Prize in 2010.

[78] N. Kuhnert , Angew. Chem. Int. Ed. 2002, 41, 1863–1864; 10.1002/1521-3773(20020603)41:11<1863::aid-anie1863>3.0.co;2-l 19750616

[79] C. O. Kappe , B. Pieber , D. Dallinger , Angew. Chem. Int. Ed. 2013, 52, 1088–1094; 10.1002/anie.201204103 23225754

[80] In 2009, our group had developed technology that made it possible to rapidly evaluate whether an observed enhancement/effect experienced in a microwave-assisted chemical transformation is the result of a purely (bulk) thermal phenomenon, or whether specific or nonthermal microwave effects are involved. Key to this protocol was the use of a microwave reaction vessel produced from silicon carbide (SiC) ceramic, which, owing to the high microwave absorptivity of SiC, shields the contents of the reaction vessel from the electromagnetic field. Used in combination with a dedicated microwave reactor with an internal FO temperature probe, this in essence allows mimicking a conventionally heated autoclave experiment under carefully controlled reaction conditions. A simple change from a nearly microwave transparent glass (Pyrex) to a strongly microwave absorbing SiC reaction vial in the same microwave reactor platform then allows to investigate the influence of the electromagnetic field on the particular chemical transformation and thus to distinguish between thermal and specific/nonthermal microwave effects. The development of this technology and its significance for the investigation of microwave effects has been described in: C. O. Kappe , Acc. Chem. Res. 2013, 46, 1579–1585.23463987

[81] The only exception found in our laboratories relates to the use of zerovalent metals suspended in weakly microwave absorbing organic solvents. The effects seen in these instances are due to exceedingly high temperatures caused by arcing phenomena on the metal surface: B. Gutmann , A. M. Schwan , B. Reichart , C. Gspan , F. Hofer , C. O. Kappe , Angew. Chem. Int. Ed. 2011, 50, 7636–7674;

[82] A comprehensive list of all our publications in the field of microwave chemistry (∼200 original research articles, 20 reviews and 25 books/book chapters), highlighting those where comparison experiments between conventional heating and microwave heating were performed is given in the Supporting Information.

[83] An additional indication that microwave-assisted organic synthesis is based on purely thermal phenomena can be derived from the fact that it is possible to translate these processes to continuous flow processes, using *conventionally heated* flow reactors. For details, see: T. N. Glasnov , C. O. Kappe , Chem. Eur. J. 2011, 17, 11956–11968.21932289

[84] M. R. Rosana , Y. Tao , A. E. Stiegman , G. B. Dudley , Chem. Sci. 2012, 3, 1240–1246.

[85] E. Richards, “Magical microwave effects revived. Microwaves can accelerate reactions without heating”, *Chem. World* **2012**, *9*(3) 25. I note with interest that the title of this article as it appeared in print in 2012 is not the same as in the online version available now: “Magical microwaves. When a reaction speeds up in a microwave, is it down to the heat or the microwaves?”

[86] For an account on the state-of-affairs in the microwave chemistry field at the time, including a discussion on the existence of microwave effects, see:

[86a] S. K. Ritter , Chem. Eng. News 2012, 90, 32–34.

[86b] A. M. Thayer , Chem. Eng. News 2012, 90, 12–17.

[87] It took seven independent referee reports and multiple cycles of rebuttals to get our article (ref. 79) published, despite strong resistance from some referees. For the public debate that ensued, see:

[87a] G. B. Dudley , A. E. Stiegman , M. R. Rosana , Angew. Chem. Int. Ed. 2013, 52, 7918–7923; 10.1002/anie.201301539 23824983

[87b] C O. Kappe , Angew. Chem. Int. Ed. 2013, 52, 7924–7928; 10.1002/anie.201304368 23825030

[87c] S. K. Ritter , Chem. Eng. News 2014, 92, 26–28.

[88] For a study on open-vessel microwave heating from our laboratories addressing this point, see: D. Dallinger , M. Irfan , A. Suljanovic , C. O. Kappe , J. Org. Chem. 2010, 75, 5274–5288.

[89] For a recent summary of hypotheses regarding the existence of microwave effects in organic synthesis that cannot be rationalized by bulk temperature effects, see: G. B. Dudley , A. E. Stiegman , Chem. Rec. 2018, DOI: 10.1002/tcr.201700044.

[90] The Masterwave Benchtop Reactor was launched in 2010 and was developed with input from process chemists from Novartis and AstraZeneca. For details, see: D. Dallinger , H. Lehmann , J. D. Moseley , A. Stadler , C. O. Kappe , Org. Process Res. Dev. 2011, 15, 841–854.

[91] In parallel to activities within the CDLMC we entered a collaboration with BASF (2008–2010) on the scale-up of microwave-assisted transformations. For some selected examples of these results mainly involving transition metal-catalyzed cross-couplings (involving Mostafa Baghbanzadeh and Michael Fuchs in my group), see:

[91a] M. Baghbanzadeh , C. Pilger , C. O. Kappe , J. Org. Chem. 2011, 76, 1507–1510. 21250707 10.1021/jo1024464

[91b] M. Fuchs , W. Goessler , C. Pilger , C. O. Kappe , Adv. Synth. Catal. 2010, 352, 323–328.

[91c] M. Baghbanzadeh , C. Pilger , C. O. Kappe , J. Org. Chem. 2011, 76, 8138–8142. 21851080 10.1021/jo201516v

[92] The CEM Voyager CF (continuous flow) instrument was purchased in 2003 (see ref. 44) and a CEM Voyager SF (stop flow) from the CDLMC grant in 2007 (see ref. 71). See ref. 28b for details on both instruments.

[93] In a joint publication with Clariant (Germany) the use of large scale continuous flow microwave reactors (cylindrical reaction tubes of 1×60 cm) was evaluated. Here, the volumetric heating and energy savings appear to make this technology viable for manufacturing purposes where high reaction temperatures are required. For details, see: R. Morschhäuser , M. Krull , C. Kayser , C. Boberski , R. Bierbaum , P. A. Püschner , T. N. Glasnov , C. O. Kappe , Green Process. Synthesis 2012, 1, 281–290.

[94] These investigations are partially described in ref. 83 and formed the basis of a subsequent Christian Doppler Laboratory at the University of Graz focusing entirely on flow chemistry (CDLFC, 2013–2015), and ultimately laid the foundation for our current research activities in the *Center for Continuous Flow Synthesis and Processing* (CC FLOW) in Graz. See http://goflow.at for details.

[95] L. Pisani , H. Prokopcova , J. M. Kremsner , C. O. Kappe , J. Comb. Chem. 2007, 9, 415–421. 17341121 10.1021/cc0700041

[96] As described in detail in ref. 25, the fact that SiC has an extremely high thermal conductivity (350 W/mK, ∼100 times higher compared to glass) enables rapid and gradient-free heating of these microtiter plates (and of their contents) also by simply placing them on a standard hotplate. This microwave-free method using, e. g., the set-up shown in Figure 12b, is now used routinely in our laboratory for rapidly optimizing chemical transformations in parallel under sealed vessel conditions.

[97] For an early review on parallel microwave chemistry highlighting some of the problems, see: M. Matloobi , C. O. Kappe , Comb. Chem. High Throughput Screening 2007, 10, 735–750. 10.2174/138620707783018496 18478956

[98] The technology and manifold applications of the SiC microtiter plate concept are summarized in the following review: C. O. Kappe , M. Damm , Mol. Diversity 2012, 16, 5–25. 10.1007/s11030-011-9346-x 22127640

[99] D. Obermayer , B. Gutmann , C. O. Kappe , Angew. Chem. Int. Ed. 2009, 48, 8321–8324; 10.1002/anie.200904185 19784993

[100] C. O. Kappe, D. Obermayer, *Autoclave with Electrically Heatable Wall*, AT 514562 (B1), WO 2015021491 (A1), **2013**.

[101] D. Obermayer , M. Damm , C. O. Kappe , Chem. Eur. J. 2013, 19, 15827–15830. 24130085 10.1002/chem.201303638

[102] D. Obermayer , D. Znidar , G. Glotz , A. Stadler , D. Dallinger , C. O. Kappe , J. Org. Chem. 2016, 81, 11788–11801. 27934447 10.1021/acs.joc.6b02242

[103] Regrettably, progressing from prototype to commercial instrument, the original (and patented) design concept of using self-heating reaction vessels made out of semiconducting SiC ceramic had to be dropped.

[104] The instrument originally launched as Monowave 300 in 2009 (Figure 10), was subsequently developed into an instrument called Monowave 400 and is now part of a series of single-mode microwave instruments from Anton Paar (Monowave 200, 400 and 450).

[105] With the aid of S. Shaun Murphree, a visiting professor in the group under the Fulbright Scholar Program in 2007, we have established a practical lab course for MAOS that, in different incarnations, has been part of our teaching efforts at University of Graz for many years. For details, see:

[105a] S. S. Murphree , C. O. Kappe , J. Chem. Educ. 2009, 86, 227–229.

[105b] C. O. Kappe , D. Dallinger , S. S. Murphree , Practical Microwave Synthesis for Organic Chemists – Strategies, Instruments, and Protocols; Wiley-VCH, Weinheim, 2009.

[106] In this context, I want to acknowledge the contributions of several PhD students during this period that were not funded by the CDLMC and mainly pursued MAOS projects unrelated to the topics of this grant: Mitra Matloobi, Hana Prokopcova, Jamshed Hashim, Tahseen Razzaq, Irfan Muhammed, Nuzhat Arshad and Seyed Mojtaba Mirhoseini Moghaddam. The results of this research from the period 2006–2012 (an update to ref. 39 covering the 2002–2005 period) have been summarized in: D. Dallinger, C. O. Kappe, in “Seminars in Organic Synthesis, XXXVIII ”A. Corbella“ Summer School”, Societa Chimica Italiana, **2013**, p. 69–92,

[107a] R. O. M. A. de Souza , O. A. C. Antunes , W. Kroutil , C. O. Kappe , J. Org. Chem. 2009, 74, 6157–6162. 19601570 10.1021/jo9010443

[107b] A. M. Balu , D. Dallinger , D. Obermayer , J. M. Campelo , A. A. Romero , D. Carmona , F. Balas , J. Santamaria , K. Yohida , P. L. Gai , C. Vargas , C. O. Kappe , R. Luque , Green Chem. 2012, 14, 393–402.

[108] It should be noted that MAOS to some extent has become a standard tool in many laboratories, in particular in industrial medicinal chemistry labs. The use of microwave technology for performing organic synthesis, in many instances, is therefore no longer reflected in the title, abstract or the keywords of a publication. A comparison of the results obtained via a standard keyword search as described in Figure 14 for microwave publications during 2017 in *The Journal of Organic Chemistry* and a full text search makes this discrepancy clear. While the keyword search provides only 20 hits, the full text search leads to 62 genuine publications where microwave technology has been used for organic synthesis.

[109] In contrast to the field of organic synthesis, microwave technology is used successfully on large scale in other areas, such as food processing, the vulcanization of rubber, for sintering ceramics and the mining industry.

[110] In 2008, Jonathan D. Moseley, at that time a process chemist from AstraZeneca was quoted in saying that (see ref. 55b): “Virtually all new compounds now have their first synthesis in a microwave”.

[111] In comparing the number of publications in 2017 in *The Journal of Organic Chemistry* that involve microwave-assisted organic synthesis from a full text search (see ref. 108) with the overall number of papers published in the journal the same year (62 versus 1.487) this becomes very obvious. It is needless to say that not all publications in organic chemistry journals deal with synthetic procedures that are amenable to microwave heating, but a large percentage probably would be.

[112] On a more personal note and as far as equipment is concerned, I feel somewhat saddened that most experiments in microwave chemistry run today are still performed in systems that use external IR sensors for temperature measurement, despite all the research that we and others have performed that demonstrates how unreliable this method can be (see Section 5.2).

[113] An alternative and very plausible viewpoint simply is that there is little more to do fundamental research on, since basically it is understood how microwave chemistry works and a variety of instruments and technology platforms are available to apply microwave heating to a wide range of different applications, while the problem of scale-up has been resolved by a different technology (continuous processing).

[114] This expression has been used many times in the literature pertaining to the future role and penetration of microwave reactors (including by myself as quoted in ref. 55a), however, was originally coined by the late Ajay K Bose in 1997 (ref. 11a, see also ref. 86).

[115] In the context of curiosity-driven versus applied research and the often meaningless distinction between the two the reader is referred to the following essays:

[115a] G. M. Whitesides , Angew. Chem. Int. Ed. 2015, 54, 3196–3209; 10.1002/anie.201410884 25682927

[115b] G. M. Whitesides , Angew. Chem. Int. Ed. 2018, 57, 4126–4129; 10.1002/anie.201800684 29359463

