# Supplementary material for: My Twenty Years in Microwave Chemistry: From Kitchen Ovens to Microwaves that aren't Microwaves
Source: Chem Rec. 2018 Jun 15;19(1):15–39. doi: 10.1002/tcr.201800045 (PMC6391988; doi:10.1002/tcr.201800045)
Supplement: Supplementary file 1 — Supplementary [file TCR-19-15-s001.pdf]

My Twenty Years in Microwave Chemistry: From Kitchen Ovens to Microwaves that aren't Microwaves

C. Oliver Kappe*© 2018 The Authors. Published by Wiley-VCH Verlag GmbH & Co. KGaA. This is an open access article under the terms of the Creative Commons Attribution License, which permits use, distribution and reproduction in any medium, provided the original work is properly cited.

 © 2019 The Authors. Published by The Chemical Society of Japan & Wiley-VCH Verlag GmbH & Co. KGaA

Supporting Information

List of Microwave Chemistry Publications from the Kappe Laboratory (1999-2017)

All publications involving microwave experiments are listed. Publications that focus on the investigations of microwave effects and/or involve detailed comparison experiments between conventional and microwave heating experiments are marked with an asterisk*

1) Original Research Articles (Science Citation Index):

a) Early Publications Using Domestic Microwave Ovens (1999-2001)

1. Microwave-Assisted High-Speed Parallel Synthesis of 4-Aryl-3,4-dihydropyrimidin-2(1H)-ones using a Solventless Biginelli Condensation Protocol.
C. O. Kappe, D. Kumar, R. S. Varma,
Synthesis, **1999**, 1799-1803.

2. X-Ray Structure, Conformational Analysis, Enantioseparation, and Determination of Absolute Configuration of the Mitotic Kinesin Eg5 Inhibitor Monastrol.
C. O. Kappe, O. V. Shishkin, G. Uray, P. Verdino,
Tetrahedron **2000**, *56*, 1859-1862.

3. Design and Synthesis of a Conformationally Rigid Mimic of the Dihydropyrimidine Calcium Channel Modulator SQ 32,926.
B. Jauk, T. Pernat, C. O. Kappe,
Molecules **2000**, *5*, 227-239.

4. Microwave-Mediated Regioselective Synthesis of Novel Pyrimido[1,2-a]pyrimidines under Solvent-free Conditions.
J. J. Vanden Eynde, N. Hecq, O. Kataeva, C. O. Kappe,
Tetrahedron **2001**, *57*, 1785-1791.

b) Early Publications Using Multimode Technology (MLS /Milestone ETHOS, 2000-2003)

5.* Microwave-Mediated Biginelli Reactions Revisited. On the Nature of Rate and Yield Enhancements.
A. Stadler, C. O. Kappe,
J. Chem. Soc., Perkin Trans. 2 **2000**, 1363-1368.

6.* High-Speed Couplings and Cleavages in Microwave-Heated, Solid-Phase Reactions at High Temperatures.
A. Stadler, C. O. Kappe,
Eur. J. Org. Chem. **2001**, 919-925.

7. The Effect of Microwave Irradiation on Carbodiimide-Mediated Esterifications on Solid-Support.
A. Stadler, C. O. Kappe,
Tetrahedron **2001**, *57*, 3915-3920.

8. Rapid Parallel Synthesis of Polymer-Bound Enones Utilizing Microwave-Assisted Solid-Phase Chemistry.
G. A. Strohmeier, C. O. Kappe,
J. Comb. Chem. **2002**, *4*, 154-161.

9. Microwave-Assisted Reactions under Open and Closed Vessel Conditions. A Case Study.
A. Stadler, S. Pichler, G. Horeis, C. O. Kappe,
Tetrahedron **2002**, *58*, 3177-3183.

c) Early Publications Using Single-Mode Technology (Personal Chemistry Smith/Emrys Synthesizer, 2001-2005)

10. Automated Library Generation Using Sequential Microwave-Assisted Chemistry. Application Toward the Biginelli Multicomponent Condensation.
A. Stadler, C. O. Kappe,
J. Comb. Chem. **2001**, *3*, 624-630.

11. High-Speed Microwave-Promoted Mitsunobu Inversions. Application to the Deracemization of Sulcatol.
A. Steinreiber, A. Stadler, S. F. Mayer, K. Faber, C. O. Kappe,
Tetrahedron Lett. **2001**, *42*, 6283-6286.

12. Traceless Solid Phase Synthesis of Bicyclic Dihydropyrimidones Using Multidirectional Cyclization Cleavage.
R. Pérez, T. Beryozkina, O. I. Zbruyev, W. Haas, C. O. Kappe,
J. Comb. Chem. **2002**, *4*, 501-510.

13. A Tandem Intramolecular Michael-Addition/Elimination Sequence in Dihydropyrimidone to Quinoline Rearrangements
N. Stiasni, C. O. Kappe,
ARKIVOC **2002**, (8), 71-79.

14. Rapid Formation of Triarylphosphines by Microwave-Assisted Transition Metal-Catalyzed C-P Cross-Coupling Reactions.
A. Stadler, C. O. Kappe,
Org. Lett. **2002**, *4*, 3541-3543.

15. High-Speed Microwave-Promoted Hetero-Diels-Alder Reactions of 2(1H)-Pyrazinones in Ionic Liquid Doped Solvents.
E. Van der Eycken, P. Appukkuttan, W. De Borggraeve, W. Dehaen, D. Dallinger, C. O. Kappe,
J. Org. Chem. **2002**, *67*, 7904-7907.

16.* Microwave-Assisted Ring-Closing Metathesis Revisited. On the Question of the Non-Thermal Microwave Effect.
S. Garbacia, B. Desai, O. Lavastre, C. O. Kappe,
J. Org. Chem. **2003,** *68,* 9136-9139.

17. Rapid Preparation of Thioamide Building Blocks via Microwave-Promoted Three-Component Kindler Reactions.
O. I. Zbruyev, N. Stiasni, C. O. Kappe,
J. Comb. Chem. **2003**, *5*, 145-148.

18. High-Throughput Synthesis of N3-Acylated Dihydropyrimidines Combining Microwave-Assisted Synthesis and Scavenging Techniques.
D. Dallinger, N. Yu. Gorobets, C. O. Kappe,
Org. Lett. **2003**, *5*, 1205-1208.

19. A Three-Component Synthesis of Pyrido[2,3-d]pyrimidines.
N. Mont, J. Teixidó, J. I. Borrell, C. O. Kappe,
Tetrahedron Lett. **2003**, *44*, 5385-5387.

20. An Exploratory Study on Microwave-Assisted Solid-Phase Diels-Alder Reactions of 2(1H)-Pyrazinones: The Elaboration of a New Tailor-Made Acid-Labile Linker.
N. Kaval, J. Van der Eycken, J. Caroen, W. Dehaen, G. A. Strohmeier, C. O. Kappe, E. Van der Eycken,
J. Comb. Chem. **2003**, *5*, 560-568.

21. Microwave-Enhanced Transition Metal-Catalyzed Decoration of 2(1H)-Pyrazinone Scaffolds.
N. Kaval, K. Bisztray, W. Dehaen, C.O. Kappe, E. Van der Eycken,
Mol. Diversity **2003**, *7,* 125-133.

22. A One-Pot Microwave-Assisted Synthesis of Pyrido[2,3-d]pyrimidines.
N. Mont, J. Teixidó, C. O. Kappe, J. I. Borrell,
Mol. Diversity **2003**, *7*, 153-159.

23. Microwave-Assisted Scavenging of Electrophiles Utilizing Polymer-Supported Sequestration Reagents. Application to the Synthesis of N3-Acylated Dihydropyrimidine Libraries.
D. Dallinger, N. Yu. Gorobets, C. O. Kappe,
Mol. Diversity **2003**, *7*, 229-245.

24. Microwave-Assisted Negishi and Kumada Cross-Coupling Reactions of Aryl Chlorides.
P. Walla, C. O. Kappe,
Chem. Commun. **2004,** 564-565.

25. Tunable Carbon-Carbon and Carbon-Sulfur Cross-Coupling of Boronic Acids with 3,4-Dihydropyrimidine-2-thiones.
A. Lengar, C. O. Kappe,
Org. Lett. **2004**, *6*, 771-774.

26. Rapid Microwave-Assisted Solution Phase Synthesis of Substituted 2-Pyridone Libraries.
N. Yu. Gorobets, B. H. Yousefi, F. Belaj, C. O. Kappe,
Tetrahedron **2004**, *60,* 8633-8644.

27. Stereoconservative Negishi Arylation and Alkynylation as an Efficient Approach to Enantiopure 2,2'-Diarylated 1,1'-Binaphthyls.
K. Krascsenicsováa, P. Walla, P. Kasáka, G. Uray, C. O. Kappe, M. Putala,
Chem. Commun. **2004**, 2606-2607.

28. Combining Biginelli Multicomponent and Click Chemistry: Generation of 6-(1,2,3-Triazol-1-yl)-Dihydropyrimidone Libraries.
B. Khanetskyy, D. Dallinger, C. O. Kappe,
J. Comb. Chem. **2004**, *6*, 884-892.

29. Microwave-Assisted Multistep Synthesis of Functionalized 4-Arylquinolin-2(1H)-ones using Palladium-Catalyzed Cross-Coupling Chemistry.
T. N. Glasnov, W. Stadlbauer, C. O. Kappe,
J. Org. Chem. **2005**, *70*, 3864-3870.

30. The Application of "Click Chemistry" for the Decoration of the 2(1*H*)-Pyrazinone Scaffold: Generation of New Interesting Templates.
N. Kaval, D. Ermolat'ev, P. Appukkuttan, W. Dehaen, C. O. Kappe, E. Van der Eycken,
J. Comb. Chem. **2005**, *7*, 490-502.

31. Novel Microwave-Enhanced and Metal-Catalyzed Functionalizations of the 4-Aryl-Dihydropyrimidone Template.
J. Wannberg, D. Dallinger, C. O. Kappe, M. Larhed,
J. Comb. Chem. **2005**, *7*, 574-583.

32. Porphyrins in Diels-Alder Reactions. Improvements on the Synthesis of a Barrelene-Fused Chlorin using Controlled Microwave Irradiation.
A. M. G. Silva, A. C. Tomé, M. G. P. M. S. Neves, J. A. S. Cavaleiro, C. O. Kappe,
Tetrahedron Lett. **2005**, *46*, 4723-4726.

33. Heterogeneous Hydrogenation Reactions Using a Continuous Flow High Pressure Device.
B. Desai, C. O. Kappe,
J. Comb. Chem. **2005**, *7*, 641-643.

d) Publications Using Multimode Technology (Anton Paar Synthos 3000, 2003-2005)

34. Scalability of Microwave-Assisted Organic Synthesis. From Single-Mode to Multimode Parallel Batch Reactors.
A. Stadler, B. H. Yousefi, D. Dallinger, P. Walla, E. Van der Eycken, N. Kaval, C. O. Kappe,
Org. Process Res. Dev. **2003**, *7*, 707-716.

35. The Effect of Pressure on Microwave-Enhanced Diels–Alder Reactions. A Case Study.
N. Kaval , W. Dehaen , C. O. Kappe, E. Van der Eycken,
Org. Biomol. Chem. **2004**, *2,* 154-156.

36. Microwave-Assisted Organic Synthesis in Near-Critical Water at 300 °C. A Proof-of-Concept Study.
J. M. Kremsner, C. O. Kappe,
*Eur. J. Org. Chem.***2005**, 3672-3679.

e) Publications Using Single- and Multimode Technology (Biotage, CEM, Anton Paar, Milestone, 2006-2017)

i) Organic Synthesis and Catalysis

37. Symmetrical Bisquinolones via Metal-Catalyzed Cross-Coupling and Homocoupling Reactions.
J. Hashim, T. N. Glasnov, J. M. Kremsner, C. O. Kappe,
J. Org. Chem. **2006**, *71*, 1707-1710.

38. Microwave-assisted Solution Phase Synthesis of Dihydropyrimidine C5 Amides and Esters.
B. Desai, D. Dallinger, C. O. Kappe,
Tetrahedron **2006**, *62*, 4651-4664.

39. Microwave-assisted Dimroth Rearrangement of Thiazines to Dihydropyrimidinethiones: Synthetic and Mechanistic Aspects.
T. N. Glasnov, D. J. Vugts, M. M. Koningstein, B. Desai, W. M. F. Fabian, R. V. A. Orru, C. O. Kappe,
QSAR Comb. Sci. **2006**, *25*, 509-518.

40. Microwave-Assisted Three-Component Synthesis of 7-Aryl-2-alkylthio-4,7-dihydro-1,2,4-triazolo[1,5-a]-pyrimidine-6-carboxamides and Their Selective Reduction.
V. A. Chebanov, E. A. Muravyova, S. M. Desenko, V. I. Musatov, I. V. Knyazeva, S. V. Shishkina, O. V. Shishkin, C. O. Kappe,
J. Comb. Chem. **2006**, *8*, 427-434.

41. Synthesis of 5-Aroyl-dihydropyrimidinones via Liebeskind-Srogl Thiol Ester-Boronic Acid Cross Couplings.
H. Prokopcova, L. Pisani, C. O. Kappe,
Synlett **2007**, 43-46.

42. Cyclocondensation Reactions of 5-Aminopyrazoles, Pyruvic Acids and Aldehydes. Multicomponent Approaches to Pyrazolopyridines and Related Products.
V. A. Chebanov, Y. I. Sakhno, S. M. Desenko, V. N. Chernenko, V. I. Musatov, S. V. Shishkina, O. V. Shishkin, C. O. Kappe,
Tetrahedron **2007**, 63, 1229-1242.

43. Desulfitative Carbon–Carbon Cross-Coupling of Thioamide Fragments with Boronic Acids.
H. Prokopcova, C. O. Kappe,
Adv. Synth. Catal. **2007**, *349*, 448-452.

44. Rapid Preparation of the Mitotic Kinesin Eg5 Inhibitor Monastrol Using Controlled Microwave-Assisted Synthesis.
D. Dallinger, C. O. Kappe,
Nature Protocols **2007**, *2*, 317-321.

45. Rapid Preparation of Pyranoquinolines using Microwave Dielectric Heating in Combination with Fractional Product Distillation.
T. Razzaq, C. O. Kappe,
Tetrahedron Lett. **2007**, 48, 2513-2517.

46. One-Pot, Multicomponent Route to Pyrazoloquinolizines.
V. A. Chebanov, V. E. Saraev, S. M. Desenko, V. N. Chernenko, S. V. Shishkina, O. V. Shishkin, K. M. Kobzar, C. O. Kappe,
Org. Lett. **2007**, *10*, 1691-1694.

47. 5-Aroyl-3,4-dihydropyrimidin-2-one Library Generation via Automated Sequential and Parallel Microwave-assisted Synthesis Techniques.
L. Pisani, H. Prokopcova, J. M. Kremsner, C. O. Kappe,
J. Comb. Chem. **2007**, *9*, 415-421.

48. Parallel Synthesis of an Amide Library Based on the 6,8-Dioxa-3-azabicyclo[3.2.1]octane Scaffold by Direct Aminolysis of Methyl Esters.
F. Machetti, I. Bucelli, G. Indiani, C. O. Kappe, A. Guarna,
J. Comb. Chem. **2007**, *9*, 454-461.

49. Palladium(0)-Catalyzed, Copper(I)-Mediated Coupling of Boronic Acids with Cyclic Thioamides. Selective Carbon-Carbon Bond Formation for the Functionalization of Heterocycles.
H. Prokopcova, C. O. Kappe,
J. Org. Chem. **2007**, *72*, 4440-4448.

50. Automated Generation of a Dihydropyrimidine Compound Library Using Microwave-Assisted Processing
D. Dallinger, C. O. Kappe,
Nat. Protoc. **2007**, *2*, 1713-1721.

51. Microwave-Assisted Selective 5´-*O*-Trityl Protection of Inosine Derivatives.
E. Casanova, M.-J. Pérez-Pérez, C. O. Kappe,
Synlett **2007**, 1733-1735.

52. An Algorithm for the Deconvolution of Mass Spectrosopic Patterns in Isotope Labeling Studies. Evaluation for the Hydrogen-Deuterium Exchange Reaction in Ketones.
C. C. Gruber, G. Oberdorfer, C. V. Voss, J. M. Kremsner, C. O. Kappe, W. Kroutil,
J. Org. Chem. **2007**, *72*, 5778-5783.

53. Microwave-Assisted Arylation of rac-(*E*)-3-Acetoxy-1,3-diphenylprop-1-ene with Arylboronic acids.
V. Poláčková, Š. Toma, C. O. Kappe,
Tetrahedron **2007**, *63,* 8742-8745.

54. Microwave Accelerated Aza-Claisen Rearrangements.
J. Gonda, M. Martinková, A. Zadrošová, M. Šoteková, J. Raschmanová, P. Čonka, E. Gajdošíková, C. O. Kappe,
Tetrahedron Lett. **2007**, 48, 6912-6915.

55. Synthesis of Symmetrical Bisquinolones via Nickel(0)-Catalyzed Homocoupling of 4-Chloroquinolones .
J. Hashim, C. O. Kappe,
Adv. Synth. Catal. **2007**, *349*, 2353-2360.

56. Multicomponent Cyclocondensations of ß-Ketosulfones with Aldehydes and Aminoazole Building Blocks.
E. S. Gladkov, V. A. Chebanov, S. M. Desenko, O. V. Shishkin, S. V. Shishkina, D. Dallinger, C. O. Kappe,
Heterocycles **2007**, *73,* 469-480.

57. Microwave-Assisted Click Chemistry for the Preparation of 3- and 4-Triazolyl-2(1*H*)-quinolones as Potential Fluorescent Probes.
T. N. Glasnov, C. O. Kappe,
QSAR Comb. Sci. **2007**, *26*, 1261-1265.

58. Microwave-Assisted Catalyst-Free Transesterification of Triglycerides with 1-Butanol under Supercritical Conditions.
J. Geuens, J. M. Kremsner, B. A. Nebel, S. Schober, R. A. Dommisse, M. Mittelbach, S. Tavernier, C. O. Kappe, B. U. W. Maes,
Energy Fuels **2008**, *22,* 643-645.

59. Integration of High Speed Microwave Chemistry and a Statistical "Design of Experiment" Approach for the Synthesis of the Mitotic Kinesin Eg5 Inhibitor Monastrol.
T. N. Glasnov, H. Tye, C. O. Kappe,
Tetrahedron **2008**, *64*, 2035-2041.

60. Synthesis of Bisquinolone-Based Mono- and Diphosphine Ligands of the Aza-BINAP Type.
N. Arshad, J. Hashim. C. O. Kappe,
J. Org. Chem. **2008**, *73,* 4755-4758.

61. Tuning of Chemo- and Regioselectivities in Multicomponent Condensations of 5-Aminopyrazoles, Dimedone and Aldehydes.
V. Chebanov, V. Saraev, S. Desenko, V. Chernenko, I. Knyazeva, U. Groth, T. Glasnov, C. O. Kappe
J. Org. Chem. **2008**, *73,* 5110-5118.

62. Microwave-Assisted One-Pot Diboration/Suzuki Cross-Couplings. A Rapid Route to Tetrasubstitued Alkenes.
H. Prokopcova, J. Ramírez, E. Fernández, C. O. Kappe,
Tetrahedron Lett. **2008**, 49, 4831-4835.

63. Multicomponent Cyclocondensation Reactions of Aminoazoles, Arylpyruvic Acids and Aldehydes with Controlled Chemoselectivity.
Y. I. Sakhno, S. M. Desenko, S. V. Shishkina, O. V. Shishkin, D. O. Sysoyev, U. Groth, C. O. Kappe, V. A. Chebanov
Tetrahedron **2008**, *64*, 11041-11049

64.* Heterogeneous Versus Homogeneous Palladium Catalysts for Ligandless Mizoroki-Heck Reactions. A Comparison of Batch/Microwave and Continuous Flow Processing.
T. N. Glasnov, S. Findenig, C. O. Kappe
Chem. Eur. J. **2009**, *15*, 1001-1015.

65. Microwave-Assisted Aliphatic Fluorine–Chlorine Exchange Using Triethylamine Trihydrofluoride (TREAT–HF).
J. M. Kremsner, M. Rack, C. Pilger, C. O. Kappe,
Tetrahedron Lett. **2009**, *50*, 3665-3668.

66. Bisquinolones as Chiral Fluorophores – A Combined Experimental and Computational Study of Absorption and Emission Characteristics
G. Uray, A.-M. Kelterer, J. Hashim, T. N. Glasnov, C. O. Kappe, W. M. F. Fabian,
J. Mol. Struct. **2009**, *929*, 85-96.

67. One-Pot Multicomponent Preparation of Tetrahydropyrazoloquinolinones and Tetrahydropyrazoloquinazolinones.
T. N. Glasnov, C. O. Kappe,
Org. Synth. **2009**, *86*, 252-261.

68.* Microwave-Assisted Cross-Coupling and Hydrogenation Chemistry Using Heterogeneous Transition-Metal Catalysts: An Evaluation of the Role of Selective Catalyst Heating.
M. Irfan, M. Fuchs, T. N. Glasnov, C. O. Kappe
Chem. Eur. J. **2009**, *15*, 11608-11618.

69. High-Speed Microwave-Assisted Synthesis of the Trifluoromethylpyrazol-derived Canonical Transient Receptor Potential Channel (TRPC3) Inhibitor Pyr3.
T. N. Glasnov, K. Groschner, C. O. Kappe
ChemMedChem **2009**, *4*, 1816-1818.

70. Palladium(0)-Catalyzed, Copper(I)-Mediated Coupling of Cyclic Thioamides with Alkenylboronic Acids, Organostannanes and Siloxanes.
N. Arshad, J. Hashim. C. O. Kappe,
J. Org. Chem. **2009**, *74,* 5118-5121.

71. Microwave-Assisted Synthesis of 5-Arylazo-4,6-disubstituted-3-cyano-2-pyridone Dyes.
D. Z. Mijin, M. Baghbanzadeh, C. Reidlinger, C. O. Kappe
Dyes Pigments **2010**, 85, 73-78.

72. Diversity-Oriented Synthesis of Dibenzoazocines and Dibenzoazepines via a Microwave-Assisted Intramolecular A3-Coupling Reaction.
J. B. Bariwal, D. S. Ermolat'ev, T. N. Glasnov, K. Van Hecke, V. P. Mehta, L. Van Meervelt, C. O. Kappe, E. Van der Eycken,
Org. Lett. **2010**, *13*, 2774–2777.

73. Structure-Activity Relationships and Molecular Docking of Novel Dihydropyrimidine-Based Mitotic Eg5 Inhibitors.
H.. Prokopcová, D. Dallinger, G. Uray, H. Y. K. Kaan, V. Ulaganathan, F. Kozielski, C. Laggner, C. O. Kappe,
ChemMedChem **2010**, *5*, 1760-1769.

74.* A Unified Mechanistic View on the Morita-Baylis-Hillman Reaction: Computational and Experimental Investigations.
D. Cantillo, C. O. Kappe,
J. Org. Chem. **2010**, *75,* 8615-8626.

75.* Rapid Nickel-Catalyzed Suzuki- Miyaura Cross-Couplings of Aryl Carbamates and Sulfamates Utilizing Microwave Heating.
M. Baghbanzadeh, C. Pilger, C. O. Kappe,
J. Org. Chem. **2011**, *76,* 1507-1510.

76. Microwave-Assisted Nickel(II) Acetylacetonate-Catalyzed Arylation of Aldehydes with Arylboronic Acids.
W. Chen, M. Baghbanzadeh, C. O. Kappe,
Tetrahedron Lett. **2011**, 52, 1677-1679.

77. Mechanistic Insights on Azide-Nitrile Cycloadditions: On the Dialkyltin Oxide-Trimethylsilyl Azide Route and a New Vilsmeier-Haack-Type Organocatalyst.
D. Cantillo, B. Gutmann, C. O. Kappe,
J. Am. Chem. Soc. **2011**, *133*, 4465-4475.

78. Pentafluorophenyl-bis(diethylamino)phosphane - A Push-Pull Phosphane Available for Coordination.
A. Orthaber, M. Fuchs, F. Belaj, G. N. Rechberger, C. O. Kappe, R. Pietschnig,
Eur. J. Inorg. Chem. **2011**, 2588-2596.

79. Palladium-Catalyzed Direct Arylation of Heteroaromatic Compounds: Improved Conditions Utilizing Controlled Microwave Heating.
M. Baghbanzadeh, C. Pilger, C. O. Kappe,
J. Org. Chem. **2011**, *76*, 8138-8142.

80. Direct Arylation of Benzene with Aryl Bromides Using High-Temperature/High-Pressure Process Windows – Expanding the Scope of C-H Activation Chemistry
B. Pieber, D. Cantillo, C. O. Kappe,
Chem. Eur. J. **2012**, *18*, 5047-5055.

81. Copper-Catalyzed C-O Bond Formation by Direct α-C-H Bond Activation of Ethers Using Stoichiometric Amounts of Peroxide in Batch and Continuous Flow Formats.
G. S. Kumar, B. Pieber, K. R. Reddy, C. O. Kappe,
Chem. Eur. J. **2012**, *18*, 6124-6128.

82. High-Speed Microwave-assisted Synthesis of SEA0400 – a Selective Inhibitor of the Na+/Ca2+ Exchanger.
G. Guedes de la Cruz, K. Groschner, C. O. Kappe, T. N. Glasnov
Tetrahedron Lett. **2012**, *53*, 3731-3734.

83. Novel Pyrazole Compounds for Pharmacological Discrimination Between Receptor-operated and Store-operated Ca2+ Entry Pathways.
H. Schleifer, B. Doleschal, M. Lichtenegger, R. Oppenrieder, I. Derler, I. Frischauf, T. N. Glasnov, C. O. Kappe, C. Romanin, K. Groschner
Br. J. Pharmacol. **2012**, *167*, 1712–1722.

84. An Experimental and Computational Assessment of Acid-Catalyzed Azide-Nitrile Cycloadditions.
D. Cantillo, B. Gutmann, C. O. Kappe,
J. Org. Chem. **2012**, *77*, 10882−10890.

85. A Microwave Approach to the Synthesis of certain 4-Substituted Phenyl-6-phenyl-3-cyano-2-pyridones.
A. Marinković, D. Mijin, J. Mirković, V. Maslak, C. O. Kappe,
J. Serb. Chem. Soc. **2014**, *79*, 759-765.

86. A Detailed Investigation of the Multicomponent Reaction of Salicylaldehyde, Ethyl acetoacetate and Isocyanides under Microwave Heating
T. Amanpour, K. Zangger, F. Belaj, A. Bazgir, D. Dallinger, C. O. Kappe
Tetrahedron **2015**, *71*, 7159-7169.

87. Copper/Nafion-Catalyzed Hydroarylation Process Involving Ketenimine Intermediates: A Novel and Synthetic Approach to 4-Sulfonamidoquinoline-2-ones and Derivatives Thereof
B. Reichart, G. Guedes de la Cruz, K. Zangger, C. O. Kappe, T. Glasnov,
Adv. Synth. Catal. **2016**, *358,* 50–55.

ii) Solid-Phase Organic and Peptide Synthesis

88. Rapid Solid-Phase Peptide Synthesis Using Controlled Microwave Irradiation.
B. Basca, B. Desai, G. Dibo, C. O. Kappe,
J. Pept. Sci. **2006**, *12*, 633-638.

89. Microwave-Assisted Solution- and Solid-Phase Synthesis of 2-Amino-4-arylpyrimidine Derivatives.
M. Matloobi, C. O. Kappe,
J. Comb. Chem. **2007**, *9*, 275-284.

90. Rapid Solid-Phase Synthesis of a Calmodulin-Binding Peptide Using Controlled Microwave Irradiation
B. Bacsa, C. O. Kappe,
Nat. Protoc. **2007**, *2*, 2222-2227.

91.* Solid-Phase Synthesis of Difficult Peptide Sequences at Elevated Temperatures – A Critical Comparison of Microwave and Conventional Heating Technologies.
B. Bacsa, K. Horváti, S. Bősze, F. Andreae, C. O. Kappe
J. Org. Chem. **2008**, *73,* 7532-7542.

92.* Direct Solid-Phase Synthesis of the β-Amyloid (1-42) Peptide Using Controlled Microwave Heating.
B. Bacsa, S. Bősze, C. O. Kappe
J. Org. Chem. **2010**, *75,* 2103-2106.

iii) Polymer and Starch Chemistry

93. Selectivity of EO-PO Diblock Copolymers in the Microwave-assisted Anionic Ring-Opening Polymerization of Propylene Oxide with PEG as Initiator.
M. I. Malik, B. Trathnigg, C. O. Kappe,
Macromol. Chem. Phys. **2007**, *208,* 2510-2524.

94. Microwave-Assisted Synthesis and Characterization of End-Functionalized Poly(propylene oxide) as Model Compounds.
M. I. Malik, B. Trathnigg, C. O. Kappe,
Eur. Pol. J. **2008**, *44,* 144-154.

95. Amphiphilic Polymers Based on Higher Alkylene Oxides: Synthesis and Characterization by Different Chromatographic Techniques.
M. I. Malik, B. Trathnigg, C. O. Kappe
J. Chromat. A. **2009**, *1216,* 1167-1173.

96. Microwave-Assisted Polymerizations of Higher Alkylene Oxides.
M. I. Malik, B. Trathnigg, C. O. Kappe
Eur. Pol. J. **2009**, *45,* 899-910.

97. Characterization of Poly(ethylene glycol)-b-poly(epsilon-caprolactone) by Liquid Chromatography under Critical Conditions: Influence of Catalysts and Reaction Conditions on Product Composition.
H. Ahmed, B. Trathnigg, C. O. Kappe, R. Saf,
Eur. Pol. J. **2009**, *45,* 2338-2347.

98. Synthesis of Poly(ε-caprolactone) Diols and and EO-CL Block Copolymers and their Characterization by Liquid Chromatography and MALDI-TOF-MS.
H. Ahmed, B. Trathnigg, C. O. Kappe, R. Saf,
Eur. Pol. J. **2010**, *46,* 494-505.

99. Treatment of Aqueous Waxy Corn Starch Dispersions in a Dedicated Microwave Reactor and their Hydrodynamic Properties Determined Using Asymmetrical Flow Field-Flow Fractionation.
S. Juna, A. Huber, M. Damm, C. O. Kappe,
Starch **2012**, *64*, 652-656.

100. Influence of Temperature on the Apparent Molar Masses and Sizes of Pregelatinised Wx Corn in Aqueous Media Determined Using Asymmetrical Flow Field-Flow Fractionation.
S. Juna, M. Damm, C. O. Kappe, A. Huber,
Starch **2013**, *65*, 954-961.

101. Nanoprecipitaton of Native Pea Starches Treated in Alkaline Media at various Temperatures Employing a Dedicated Microwave Reactor.
S. Juna, S. Hayden, M. Damm, C. O. Kappe, A. Huber,
Starch **2014**, *66*, 124-132.

102. Microwave Mediated Preparation of Nanoparticles from wx Corn Starch Employing Nanoprecipitation,
S. Juna, S. Hayden, M. Damm, C. O. Kappe, A. Huber,
Starch **2014**, *66,* 316-325.

iv) Nanomaterials Research

103.* Investigation of the Formation of $CuInS_2$ Nanoparticles by the Oleylamine Route – Comparison of Microwave-Assisted with Conventional Syntheses.
A. Pein, M. Baghbanzadeh, T. Rath, W. Haas, E. Maier, H. Amenitsch, F. Hofer, C. O. Kappe, G. Trimmel,
Inorg. Chem. **2011**, *50*, 193-200.

104.* A Critical Investigation on the Microwave-Assisted Preparation of Supported Iron Oxide Nanoparticles on Silica-type Mesoporous Materials.
A. M. Balu, D. Dallinger, D. Obermayer, J. M. Campelo, A. A. Romero, D. Carmona, F. Balas, J. Santamaria, K. Yohida, P. L. Gai, C. Vargas, C. O. Kappe, R. Luque,
Green Chem. **2012**, *14*, 393-402.

105.* A Critical Assessment of the Specific Role of Microwave Irradiation in the Synthesis of ZnO Micro- and Nano-structured Materials.
M. Baghbanzadeh, S. D. Škapin, Z. C. Orel, C. O. Kappe,
Chem. Eur. J. **2012**, *18*, 5724-5731.

106.* Microwave-assisted Synthesis of CdSe Quantum Dots: Can the Electromagnetic Field Influence the Formation and Quality of the Resulting Nanocrystals?
M. Mirhosseini Moghaddam, M. Baghbanzadeh, A. Keilbach, C. O Kappe,
Nanoscale **2012**, *4*. 7435-7442.

107.* In-Situ Preparation and Characterization of Silver Nanocomposites on Cellulosic Fibers - Microwave vs. Conventional Heating.
D. Breitwieser, M. Mirhosseini Moghaddam, S. Spirk, M. Baghbanzadeh, T. Pivec, H. Fasl, V. Ribitsch, C. O. Kappe,
Carbohydr. Polym. **2013**, 94, 677-686.

108.* A Critical Investigation on the Existence of Selective Microwave Absorption in the Synthesis of CdSe Quantum Dots.
M. M. Moghaddam, C. O. Kappe,
Aust. J. Chem. **2014**, *67*, 1180-1188.

109. Continuous Flow Preparation of Supported Iron Oxide Nanoparticles on Porous Silicates
A. Yepez, F. L.Y. Lam, A. A. Romero, C. O. Kappe, R. Luque,
ChemCatChem **2015**, *7*, 276–282.

v) (Bio)analytical Applications

110.* An Evaluation of Microwave-Assisted Derivatization Procedures Using Hyphenated Mass Spectrometric Techniques.
M. Damm, G. Rechberger, M. Kollroser, C. O. Kappe,
J. Chromat. A. **2009**, 31, 5875-5881.

111. Microwave-assisted High-throughput Derivatization Techniques Utilizing Silicon Carbide Microtiter Platforms.
M. Damm, G. Rechberger, M. Kollroser, C. O. Kappe,
J. Chromat. A. **2010**, *1217*, 167-170.

112.* Microwave-Assisted High-Throughput Acid Hydrolysis in Silicon Carbide Microtiter Platforms - A Rapid and Low Volume Sample Preparation Technique for Total Amino Acid Analysis in Proteins and Peptides.
M. Damm, M. Holzer, G. Radspieler, G. Marsche, C. O. Kappe,
J. Chromat. A. **2010**, 1217, 7826-7832.

113. Microwave-assisted Forced Degradation Using High-Throughput Microtiter Platforms.
B. Prekodravac, M. Damm, C. O. Kappe,
J. Pham. Biomed. Anal. **2011**, *53*, 867-873.

114.* A High-Throughput Platform for Low-Volume High-Temperature/Pressure Sealed Vessel Solvent Extractions.
M. Damm, C. O. Kappe,
Anal. Chim. Acta **2011**, *707*, 76-83.

vi) Microwave Technology and Equipment Development

115. Silicon Carbide Passive Heating Elements in Microwave-Assisted Organic Synthesis,
J. M. Kremsner, C. O. Kappe,
J. Org. Chem. **2006**, *71*, 4651-4658.

116. High-Throughput Microwave-Assisted Organic Synthesis - Moving from Automated Sequential to Parallel Library Generation Formats in Silicon Carbide Microtiter Plates.
J. M. Kremsner, A. Stadler, C. O. Kappe,
J. Comb. Chem. **2007**, *9*, 285-291.

117. High-Throughput Experimentation Platform: Parallel Microwave Chemistry in HPLC/GC Vials.
M. Damm, C. O. Kappe,
J. Comb. Chem. **2009**, *11*, 460-468.

118. Parallel Microwave Synthesis of 2-Styrylquinazolin-4(3*H*)-ones in a High-Throughput Platform Using HPLC/GC Vials as Reaction Vessels.
M. Baghbanzadeh, M. Molnar, M. Damm, C. Reidlinger, M. Dabiri, C. O. Kappe,
J. Comb. Chem. **2009**, *11*, 676-684.

119.* Parallel Microwave Chemistry in Silicon Carbide Reactor Platforms: An In-Depth Investigation Into Heating Characteristics.
M. Damm, C. O. Kappe,
Mol. Diversity **2009**, *13*, 529-543.

120. Scale-Up of Microwave-assisted Reactions in a Multimode Bench-top Reactor.
D. Dallinger, H. Lehmann, J. D. Moseley, A. Stadler, C. O. Kappe,
Org. Process Res. Develop. **2011**, *15*, 841-854.

121. Design and Evaluation of Improved Magnetic Stir Bars for Single-mode Microwave Reactors
D. Obermayer, M. Damm, C. O. Kappe,
Org. Biomol. Chem. **2013**, *11*, 4949-4956.

122.* Simulating Microwave Chemistry in a Resistance Heated Autoclave Made of Semiconducting Silicon Carbide Ceramic.
D. Obermayer, M. Damm, C. O. Kappe,
Chem. Eur. J. **2013**, *19*, 15827-15830.

123.* Design and Performance Validation of a Conductively Heated Sealed-Vessel Reactor for Organic Synthesis.
D. Obermayer, D. Znidar, G. Glotz, A. Stadler, D. Dallinger, C. O. Kappe.
J. Org. Chem. **2016**, *81*, 11788-11801.

vii) Temperature Measurements, Microwave Effects and Simulation

124.* Microwave-Assisted Asymmetric Organocatalysis. A Probe for Non-Thermal Microwave Effects and the Concept of Simultaneous Cooling.
M. Hosseini, N. Stiasni, V. Barbieri, C. O. Kappe,
J. Org. Chem. **2007**, *72*, 1417-1424.

125.* Nonthermal Microwave Effects Revisited – On the Importance of Internal Temperature Monitoring and Agitation in Microwave Chemistry.
M. A. Herrero, J. M. Kremsner, C. O. Kappe,
J. Org. Chem. **2008**, *73*, 36-47.

126.* Investigating the Existence of Nonthermal/Specific Microwave Effects Using Silicon Carbide Heating Elements as Power Modulators.
T. Razzaq, J. M. Kremsner, C. O. Kappe
J. Org. Chem. **2008**, *73*, 6321-6329.

127.* Kinetic Resolution of *rac*-1-Phenylethanol Using Immobilized Lipases – A Critical Comparison of Microwave and Conventional Heating Protocols.
R. O. M. A. de Souza, O. A. C. Antunes, W. Kroutil, C. O. Kappe,
J. Org. Chem. **2009**, *74,* 6157-6162.

128.* Microwave Chemistry in Silicon Carbide Reaction Vials: Separating Thermal from Nonthermal Effects.
D. Obermayer, B. Gutmann, C. O. Kappe
Angew. Chem. Int. Ed. **2009**, 48, 8321-8324.

129.* On the Importance of Simultaneous Infrared/Fiber-Optic Temperature Monitoring in the Microwave-Assisted Synthesis of Ionic Liquids.
D. Obermayer, C. O. Kappe
Org. Biomol. Chem. **2010**, *8,* 114-121.

130. Understanding Microwave Heating Effects in Single Mode Type Cavities – Theory and Experiment.
J. Robinson, S. Kingman, D. Irvine, P. Licence, A. Smith, G. Dimitrakis, D. Obermayer, C. O. Kappe,
Phys. Chem. Chem. Phys. **2010**, *12*, 4750-4758.

131. Electromagnetic Simulations of Microwave Heating Experiments Using Reaction Vessels Made out of Silicon Carbide.
J. Robinson, S. Kingman, D. Irvine, P. Licence, A. Smith, G. Dimitrakis, D. Obermayer, C. O. Kappe,
Phys. Chem. Chem. Phys. **2010**, *12,* 10793-10800.

132.* An Investigation of Wall Effects in Microwave-Assisted Ring-Closing Metathesis and Cyclotrimerization Reactions.
D. Dallinger, M. Irfan, A. Suljanovic, C. O. Kappe,
J. Org. Chem. **2010**, *75,* 5278–5288.

133.* Sintered Silicon Carbide: A New Ceramic Vessel Material for Microwave Chemistry in Single-Mode Reactors.
B. Gutmann, D. Obermayer, B. Reichart, B. Prekodravac, M. Irfan, J. M. Kremsner, C. O. Kappe,
Chem. Eur. J. **2010**, *16*, 12182-12194.

134.* Activation and Deactivation of a Chemical Transformation by an Electromagnetic Field – Evidence for Specific Microwave Effects in the Formation of Grignard Reagents.
B. Gutmann, A. M. Schwan, B. Reichart, C. Gspan, F. Hofer, C. O. Kappe,
Angew. Chem. Int. Ed. **2011**, *50*, 7636-7640.

135. Characterization of Microwave-induced Electric Discharge Phenomena in Metal-Solvent Mixture.
W. Chen, B. Gutmann, C. O. Kappe,
ChemistryOpen **2012**, *1*, 39-48.

136.* Can Electromagnetic Fields Influence the Structure and Enzymatic Digest of Proteins? A Critical Evaluation of Microwave-Assisted Proteomics Protocols.
M. Damm, C. Nusshold, D. Cantillo, G. N. Rechberger, K. Gruber, W. Sattler, C. O. Kappe,
J. Proteom. **2012**, *75,* 5533-5543.

137.* On the Importance of Accurate Internal Temperature Measurements in the Microwave Dielectric Heating of Viscous Systems and Polymer Synthesis.
S. Hayden, M. Damm, C. O. Kappe,
Macromol. Chem. Phys. **2013**, *214*, 423-434.

138.* A Critical Investigation on the Occurrence of Microwave Effects in Emulsion Polymerizations.
S. Hayden, A. F. H. Studentschnig, S. Schober, C. O. Kappe,
Macromol. Chem. Phys. **2014**, *215*, 2318−2326.

viii) Process Optimization for Continuous Flow Protocols

139. Palladium(0) Nanoparticles on Glass-Polymer Composite Materials as Recyclable Catalysts: A Comparison Study on Their Use in Batch and Continuous Flow Processes.
K. Mennecke, R. Cecilia, T. N. Glasnov, S. Gruhl, C. Vogt, A. Feldhoff, M. A. L. Vargas, C. O. Kappe, U. Kunz, A. Kirschning,
Adv. Synth. Catal. **2008**, *350*, 717-730.

140. Continuous Flow Microreactor Chemistry Under High Temperature/Pressure Conditions.
T. Razzaq, T. N. Glasnov, C. O. Kappe
Eur. J. Org. Chem. **2009**, 1321-1325.

141. Continuous Flow Hydrogenation of Functionalized Pyridines.
M. Irfan, E. Petricci, T. N. Glasnov, M. Taddei, C. O. Kappe
Eur. J. Org. Chem. **2009**, 1326-1334.

142. Accessing Novel Process Windows in a High-Temperature/Pressure Capillary Flow Reactor.
T. Razzaq, T. N. Glasnov, C. O. Kappe,
Chem. Eng. Technol. **2009**, *32,* 1702-1716.

143.* Translating High-Temperature Microwave Chemistry to Scalable Continuous Flow Processes.
M. Damm, T. N. Glasnov, C. O. Kappe,
Org. Process Res. Develop. **2010**, *14*, 215-224.

144.* Mechanistic Insights Into Copper(I)-Catalyzed Azide-Alkyne Cycloadditions Using Continuous Flow Conditions.
M. Fuchs, W. Goessler, C. Pilger, C. O. Kappe
Adv. Synth. Catal. **2010**, *352*, 323-328.

145. Synthesis of 5-Substituted 1*H*-Tetrazoles from Nitriles and Hydrazoic Acid Using a Safe and Scalable High-Temperature Microreactor Approach.
B. Gutmann, J.-P. Roduit, D. Roberge, C. O. Kappe
Angew. Chem. Int. Ed. **2010**, 49, 7101-7105.

146. Toward a Continuous Flow Synthesis of Boscalid.
T. N. Glasnov, C. O. Kappe,
Adv. Synth. Catal. **2010**, *352*, 3089-3097.

147. Unusual Behavior in the Reactivity of 5-Substituted-1*H*-tetrazoles in a Resistance-Heated Microreactor.
B. Gutmann, T. N. Glasnov, T. Razzaq, W. Goessler, D. M. Roberge, C. O. Kappe,
Beilstein. J. Org. Chem. **2011**, 7, 503-517.

148. A Scalable Two-Step Continuous Flow Synthesis of Nabumetone and Related 4-Aryl-2-butanones.
M. Viviano, T. N. Glasnov, B. Reichart, G. Tekautz, C. O. Kappe
Org. Process Res. Develop. **2011**, *15*, 858-870.

149. Microwave-Assisted and Continuous Flow Multistep Synthesis of 4-(Pyrazol-1-yl)carboxanilides.
D. Obermayer, T. N. Glasnov, C. O. Kappe,
J. Org. Chem. **2011**, *76*, 6657-6669.

150. Microwave-assisted Grafting to MCM-41 Silica and its Application as Catalyst in Flow Chemistry.
M. Oliverio, A. Procopio, T. N. Glasnov, W. Goessler, C. O. Kappe,
Aust. J. Chem. **2011**, *64*, 1522-1529.

151. A Two-Step Continuous Flow Synthesis of N-(2-Aminoethyl)acylamides via Ring-Opening/Hydrogenation of Oxazolines.
B. Gutmann, J.-P. Roduit, D. Roberge, C. O. Kappe,
Chem. Eur. J. **2011**, *17*, 13146–13150.

152. High-Temperature Continuous Flow Synthesis of 1,3,4-Oxadiazoles via N-Acylation of 5-Substituted Tetrazoles.
B. Reichart, C. O. Kappe,
Tetrahedron Lett. **2012**, *53*, 952-955.

153. Safe Generation and Synthetic Utilization of Hydrazoic Acid in a Continuous Flow Reactor.
B. Gutmann, J.-P. Roduit, D. Roberge, C. O. Kappe,
J. Flow Chem. **2012**, *2*,8-19.

154. Flash Flow Pyrolysis: Mimicking Flash Vacuum Pyrolysis in a High-Temperature/High-Pressure Liquid-Phase Microreactor Environment.
D. Cantillo, H. Sheibani, C. O. Kappe,
J. Org. Chem. **2012**, *77*, 2463-2473.

155. Microwave-assisted Continuous Flow Synthesis on Industrial Scale.
R. Morschhäuser, M. Krull, C. Kayser, C. Boberski, R. Bierbaum, P. A. Püschner, T. N. Glasnov, C. O. Kappe,
Green Process. Synthesis **2012**, *1*, 281-290.

156. In Situ Generated Iron Oxide Nanocrystals as Efficient and Selective Catalysts for the Reduction of Nitroarenes in Continuous Flow.
D. Cantillo, M. Baghbanzadeh, C. O. Kappe,
Angew. Chem. Int. Ed. **2012**, *51,* 10190-10193.

157. Methylation Using Dimethylcarbonate Catalysed by Ionic Liquids Under Continuous Flow Conditions.
T. N. Glasnov, J. D. Holbrey, C. O. Kappe, K. R. Seddon, T. Yan,
Green Chem. **2012**, *14*, 3071-3076.

158. Continuous Flow Synthesis of n-Alkyl Chlorides in a High-Temperature Microreactor Environment.
B. Reichart, G. Tekautz, C. O. Kappe,
Org. Process Res. Develop. **2013**, *17*, 152-156.

159. On the Fischer Indole Synthesis of 7-Ethyltryptophol- Mechanistic and Process Intensification Studies under Continuous Flow Conditions.
B. Gutmann, M. Gottsponer, P. Elsner, D. Cantillo, D. M. Roberge, C. O. Kappe,
Org. Process Res. Develop. **2013**, *17*, 294-302.

160. Nanocatalysis in Continuous Flow: Supported Iron Oxide Nanoparticles for the Heterogeneous Aerobic Oxidation of Benzyl Alcohol.
D. Obermayer, A. M. Balu, A. A. Romero, W. Goessler, R. Luque, C. O. Kappe,
Green Chem. **2013**, *15*, 1530-1537.

161. Continuous Flow Synthesis of Adipic Acid from Cyclohexene Using Hydrogen Peroxide in High-Temperature Explosive Regimes.
M. Damm, B. Gutmann, C. O. Kappe,
ChemSusChem **2013**, *6*, 978-982.

162. Hydrazine-Mediated Reduction of Nitro and Azide Functionalities Catalyzed by Highly Active and Reusable Magnetic Iron Oxide Nanocrystals.
D. Cantillo, M. Mirhosseini Moghaddam, C. O. Kappe,
J. Org. Chem. **2013**, *78*, 4530-4542.

163. Versatile Low-loaded Mechanochemically Synthesized Supported Iron Oxide Nanoparticles for Continuous Flow Alkylations
A. M. Balu, A. Pineda, D. Obermayer, A. A. Romero, C. O. Kappe, R. Luque,
RSC Advances, **2013**, *3*, 16292-16295

164. In Situ Generation of Diimide from Hydrazine and Oxygen - Transfer Hydrogenation of Olefins in Continuous Flow.
B. Pieber, S. T. Martinez, D. Cantillo, C. O. Kappe,
Angew. Chem. Int. Ed. **2013**, *52*, 10241-10244.

165. A Three Step Continuous Flow Synthesis of the Biaryl Unit of the HIV Protease Inhibitor Atazanavir.
L. Dalla-Vechia, B. Reichart, T. N. Glasnov, L. S. M. Miranda, C. O. Kappe, R. O. M. A. de Souza,
Org. Biomol. Chem. **2013**, *11*, 6806-6813.

166. Phase-Transfer Catalysis – Mixing Effects in Continuous-Flow Liquid/Liquid O- and S-Alkylation Processes.
B. Reichart, T. N. Glasnov, C. O. Kappe,
Synlett **2013**, *24*, 2393-2396.

167. Direct Preparation of Nitriles from Carboxylic Acids in Continuous Flow.
D. Cantillo, C. O. Kappe,
J. Org. Chem. **2013**, *78*, 10567-10571.

168. Homogeneous Liquid-Phase Oxidation of Ethylbenzene to Acetophenone in Continuous Flow Mode
B. Gutmann, P. Elsner, D. Roberge, C. O. Kappe,
ACS Catalysis **2013**, *3*, 2669-2676.

169. Combined Batch and Continuous Flow Procedure to the Chemo-Enzymatic Synthesis of Biaryl Moiety of Odanacatib.
R. de Oliveira Lopes, A. S. de Miranda, B. Reichart, T. Glasnov, C. O. Kappe, R. C. Simon, W. Kroutil, L. S. M. Miranda, I. C. R.Leal, R. O. M. A. de Souza,
J. Mol. Catal. B. **2014**, *104*, 101-107.

170. Chemistry of Pyrrolizidine Alkaloids Revisited – Semi-Synthetic Microwave and Continuous-Flow Approaches towards Crotalaria-Alkaloids.
S. T. Martinez, A. C. Pinto, T. Glasnov, C. O. Kappe
Tetrahedron Lett. **2014**, *55*, 4181-4184.

171. Immobilized Iron Oxide Nanoparticles as Stable and Reusable Catalysts for Hydrazine-mediated Nitro Reductions in Continuous Flow
M. Mirhosseini Moghaddam, B. Pieber, T. Glasnov, C. O. Kappe,
ChemSusChem **2014**, *7*, 3122-3131.

172. Shifting Chemical Equilibria in Flow – Efficient Decarbonylation Chemistry Driven by Annular Flow Regimes.
B. Gutmann, P. Elsner, T. Glasnov, D. M. Roberge, C. O. Kappe,
Angew. Chem. Int. Ed. **2014**, *53*, 11557-11561.

173. Benchmarking Immobilized Di- and Triarylphosphine Palladium Catalysts for Continuous Flow Cross-Coupling Reactions: Efficiency, Durability and Metal Leaching Studies
R. Greco, W. Goessler, D. Cantillo, C. O. Kappe,
ACS Catalysis **2015**, *5,* 1303-1312.

174. Nafion-H Catalyzed High-Temperature/High-Pressure Synthesis of a Triarylmethane in Continuous Flow Mode
S. Hayden, T. Glasnov, C. O. Kappe,
Chem. Eng. Technol. **2015**, *5,* 1303-1312.

175. A Sequential Ugi Multicomponent/Cu-Catalyzed Azide-Alkyne Cycloaddition Approach for the Continuous Flow Generation of Cyclic Peptoids
C. E. M. Salvador, B. Pieber, P. M. Neu, A. Torvisco, C. K. Z. Andrade, C. O. Kappe,
J. Org. Chem. **2015**, *80,* 4590-4602.

176. Process Intensified Flow Synthesis of 1*H*-4-Substituted Imidazoles: Toward the Continuous Production of Daclatasvir
P. F. Carneiro, B. Gutmann, R. O. M. A. de Souza, C. O. Kappe
ACS Sust. Chem. Eng. **2015**, *3*, 3445−3453.

177. Continuous Synthesis of Hydantoins: Intensifying the Bucherer-Bergs Reaction
J. L. Monteiro, B. Pieber, A. G. Corrêa, C. O. Kappe,
Synlett **2016**, *27*, 83-87.

178. Selective Olefin Reduction in Thebaine Using Hydrazine Hydrate and O_2 under Intensified Continuous Flow Conditions
B. Pieber, D. P. Cox, C. O. Kappe
Org. Process Res. Develop. **2016**, *20,* 376−385.

179. Towards the Synthesis of Noroxymorphone via Aerobic Palladium-Catalyzed Continuous Flow *N*-Demethylation Strategies.
B. Gutmann, P. Elsner, D. P. Cox, U. Weigl, D. M. Roberge, C. O. Kappe
ACS Sust. Chem. Eng. **2016**, *4,* 6048-6061.

180. Continuous-Flow Electrophilic Amination of Arenes and Schmidt Reaction of Carboxylic Acids Utilizing the Superacidic Trimethylsilyl Azide/Triflic Acid Reagent System
Y. Chen, B. Gutmann, C. O. Kappe
J. Org. Chem. **2016***, 81*, 9372-9380.

181. Continuous Flow Synthesis of Indoles by Pd-catalyzed Deoxygenation of 2-Nitrostilbenes with Carbon Monoxide
G. Glotz, B. Gutmann, P. Hanselmann, A. Kulesza, D. Roberge, C. O. Kappe
RSC Adv. **2017**, *7*, 10469-10478.

182. Development of a Continuous-Flow Sonogashira Cross-Coupling Protocol using Propyne Gas under Process Intensified Conditions
D. Znidar, C. Hone, P. Inglesby, A. Boyd, C. O. Kappe
Org. Process Res. Dev. **2017***, 21,* 878-884.

183. An Integrated Continuous Flow Synthesis of a Key Oxazolidine Intermediate to Noroxymorphone from Naturally Occurring Opioids
A. Mata, D. Cantillo, C. O. Kappe, *Eur. J. Org. Chem*. **2017**, *24*, 6505-6510.

184. Synthesis of Mepivacaine and its Analogues by a Continuous Flow Tandem Hydrogenation−Reductive Amination Strategy
N.S. Suveges, R. O. M. A. de Souza, B. Gutmann, C. O. Kappe
Eur. J. Org. Chem. **2017***, 24,* 6511-6517.

ix) Miscellaneous

185. On the Enery Efficiency of Microwave-Assisted Organic Reactions.
T. Razzaq, C. O. Kappe,
ChemSusChem **2008**, *1,* 123-132.

186. Microwave-Assisted Carbonyl Chemistry for the Undergraduate Laboratory.
S. S. Murphree, C. O. Kappe
J. Chem. Educ. **2009**, *86*, 227-229.

187. Can Molecular Sieves Be Used as Water Scavengers in Microwave Chemistry?
M. Baghbanzadeh, C. O. Kappe
Austr. J. Chem. **2009**, *62*, 244-249.

2) Review Articles (Science Citation Index):

1. Speeding Up Solid-Phase Chemistry by Microwave Irradiation. A New Tool for High-Throughput Synthesis.
   C. O. Kappe,
   American Laboratory, **2001**, *33*, (10), 13-19.

2. High-Speed Combinatorial Synthesis Utilizing Microwave Irradiation.
   C. O. Kappe,
   Curr. Opin. Chem.Biol. **2002**, *6*, 314-320.

3. Controlled Microwave Heating in Modern Organic Synthesis.
   C. O. Kappe,
   Angew. Chem. Int. Ed. **2004**, *43*, 6250-6284.

4. The Impact of Microwave Synthesis on Drug Discovery.
   C. O. Kappe, D. Dallinger,
   Nature Rev. Drug Discov. **2006**, *5*, 51-63.

5. The Use of Microwave Irradiation in Organic Synthesis. From Laboratory Curiosity to Standard Practice in Twenty Years,
   C. O. Kappe,
   CHIMIA **2006**, *60*, 308-312.

6. Microwave-Assisted Synthesis under Continuous Flow Conditions.
   T. N. Glasnov, C.O. Kappe,
   Macromol. Rapid Commun. **2007**, 28, 395-410.

7. Microwave Synthesis in High-Throughput Environments. Moving from Automated Sequential to Microtiter Plate Formats.
   M. Matloobi, C.O. Kappe,
   Chim. Oggi **2007**, *25*, 26-31.

8. Microwave-Assisted Synthesis Using Water as Solvent.
   D. Dallinger, C. O. Kappe,
   Chem. Rev. **2007**, *107*, 2563-2591 (Special Issue on Green Chemistry).

9. Parallel Processing of Microwave-Assisted Organic Transformations.
   M. Matloobi, C.O. Kappe,
   Comb. Chem. High Throughp. Screening **2007**, *10*, 735-750.

10. Microwave Dielectric Heating in Synthetic Organic Chemistry.
    C. O. Kappe,
    Chem. Sov. Rev. **2008**, *37*, 1127-1139.

11. Controlled Microwave Heating in Modern Organic Synthesis. Highlights from the 2004-2008 Literature.
    C. O. Kappe, D. Dallinger,
    Mol. Diversity **2009**, *13*, 71-193.

12. Click Chemistry under Non-classical Reaction Conditions.
    C.O. Kappe, E. Van der Eycken,
    Chem. Soc. Rev. **2010**, *39*, 1280-1290. (Special Issue on Click Chemistry)

13. Microwave-assisted Derivatization Procedures for Gas Chromatography/Mass Spectrometry Analysis.
    S. L. Söderholm, M. Damm, C. O. Kappe
    Mol. Diversity **2010**, *14*, 869-868.

14. A Critical Assessment of the Greenness and Energy Efficiency of Microwave-assisted Organic Synthesis.
    J. D. Moseley, C. O. Kappe
    Green Chem. **2011**, 13, 794-806.

15. The Microwave-to-Flow Paradigm: Translating High-Temperature Batch Microwave Chemistry to Scalable Continuous Flow Processes.
T. N. Glasnov, C. O. Kappe
Chem. Eur. J. **2011**, *17*, 11956-11968.

16. Microwave-Assisted Synthesis of Colloidal Inorganic Nanocrystals.
M. Baghbanzadeh, L. Carbone, P. D. Cozzoli, C. O. Kappe
Angew. Chem. Int. Ed. **2011**, *50*, 11312-11359.

17. Parallel Microwave Chemistry in Silicon Carbide Microtiter Platforms – A Review.
C. O. Kappe, M. Damm
Mol. Diversity **2012**, *16, 5-25.*

18.* Microwave Effects in Organic Synthesis – Myth or Reality?
C. O. Kappe, B. Pieber, D. Dallinger
Angew. Chem. Int. Ed. **2013**, *52*, 1088-1094.

19.* Unraveling the Mysteries of Microwave Chemistry Using Silicon Carbide Reactor Technology.
C.O. Kappe
Acc. Chem. Res. **2013**, *46*, 1579-1585.

20.* How to Measure Reaction Temperature in Microwave-heated Transformations.
C.O. Kappe
Chem. Sov. Rev. **2013**, *42*, 4977-4990.

3) Books and Book Chapters

a) Authored Books:

1. Microwaves in Organic and Medicinal Chemistry.
C. O. Kappe, A. Stadler,
1. Edition - June **2005**, XII, 410 Pages, Hardcover, Monograph,
ISBN 3-527-31210-2 - Wiley-VCH, Weinheim.

Chinese Translation, Chemical Industry Press (CIP), **2007**

2. Practical Microwave Synthesis for Organic Chemists - Strategies, Instruments, and Protocols.
C.O. Kappe, D. Dallinger, S. S. Murphree
1. Edition - November **2009**, X, 306 Pages, Hardcover, Monograph,
ISBN-10-3-527-32097-0, Wiley-VCH, Weinheim.

3. Microwaves in Organic and Medicinal Chemistry.
C. O. Kappe, A. Stadler, D. Dallinger
2. Edition - April **2012**, XV, 678 Pages, Hardcover, Monograph,
ISBN 3-527-331859 - Wiley-VCH, Weinheim.

b) Edited Books:

1. Microwave-Assisted Synthesis of Heterocycles.
E. Van der Eycken, C. O. Kappe (Eds.),
April **2006**, XIV, 290 Pages, Hardcover, Monograph (Topics in Heterocyclic Chemistry Series)
ISBN-10 3-540-30983-7 - Springer, Berlin - Heidelberg - New York.

c) Monographs and Book Chapters:

1. Microwave-Assisted Combinatorial Chemistry.
C. O. Kappe, A. Stadler,
in *"Microwaves in Organic Synthesis"*, A. Loupy (Ed.), Wiley-VCH, **2002**, Chapter 12, pp 405-433.

2. Microwave-Assisted Applications Using Supported Catalysts.
B. Desai, C. O. Kappe,
in "*Topics in Current Chemistry*", A. Kirschning (Ed.), Springer, **2004**, 177-207.

3. Microwave-Assisted Solid-Phase Synthesis
A. Stadler, C. O. Kappe
in *"Microwave-Assisted Organic Synthesis"*, P. Lidstöm, J. P. Tierney (Eds.), Blackwell, **2005**, 175-219.

4. Microwave-Assisted Combinatorial and High-Throughput Synthesis.
A. Stadler, C. O. Kappe
in *"Microwaves in Organic Synthesis"*, 2nd Edition, A. Loupy (Ed.), Wiley-VCH, **2006**, 726-787.

5. Scale-Up of Microwave-Assisted Processes.
J. M. Kremsner, A. Stadler, C. O. Kappe,
in "*Topics in Current Chemistry*", M. Larhed, K. Olofsson (Eds.), Springer, **2006**, 233-278.

6. Microwave-Assisted Chemistry
C. O. Kappe,
in "*Comprehensive Medicinal Chemistry II*", D. J. Triggle, J. B. Taylor (Eds.), Elsevier, Oxford, **2006**, Chapter 3.36, pp 837-860.

7. Adventures in Microwave-Assisted Organic Synthesis: Contributions from the Kappe Laboratory 2000-2005.
C. O. Kappe, A. Stadler, D. Dallinger, G. Strohmeier, R. Perez, O. I. Zbruyev, N. Stiasni, P. Walla, N. Gorobets, B. Yousefi, N. Mont, B. Desai, A. Lengar, K. Krascsenicsová, S. Garbacia, B. Khanetskyy, T. N. Glasnov, J. M. Kremsner, A. Gomez da Silva, B. Basca,
in "*New Methodologies and Techniques for a Sustainable Organic Chemistry*", A. Mordini, F. Faigl (Eds.), Springer, Dordrecht, **2008**, pp 225-251.

8. Microwave Susceptors.
T. Besson, C.O. Kappe,
in "*Microwaves in Organic Synthesis*", 3rd Edition, A. de la Hoz, A. Loupy (Eds.), Wiley-VCH **2013**, Chapter 7, pp 297-346.

9. Adventures in Microwave-Assisted Organic Synthesis.
D. Dallinger, C. O. Kappe,
in "Seminars in Organic Synthesis, XXXVIII "A. Corbella" Summer School", Societa Chimica Italiana, **2013**, p. 69-92, ISBN 978-88-86208-73-4.

10. The Microwave-to-Flow Paradigm: Translating Batch Microwave Chemistry to Continuous Flow Processes
C. O. Kappe
in "*Flow Chemistry*", F. Darvas, V. Hessel, G. Dorman, Eds., De Gruyter, Berlin, **2014**, Volume 1, Fundamentals, Chapter 8, pp 251-258. ISBN 978-3-111-028915-2.
